# Transposable Element Dynamics Drive the Genomic Evolution and Phenotypic Diversification of Allotetraploid Common Carp

**DOI:** 10.1002/advs.77790

**Published:** 2026-09-16

**Authors:** Shuimu Hu, Zhou Jiang, Lin Chen, Gowena Ling Yi Goh, Weijing Li, Qian He, Xintong Chen, Rui Li, Yuxuan Liu, Ning Li, Tao Zhou, Peng Xu

**Affiliations:** ^1^ State Key Laboratory of Mariculture Breeding, College of Ocean and Earth Sciences Xiamen University Xiamen China; ^2^ Fujian Key Laboratory of Genetics and Breeding of Marine Organisms College of Ocean and Earth Sciences Xiamen University Xiamen China; ^3^ Key Laboratory of Exploration and Utilization of Aquatic Genetic Resources College of Fisheries and Life Science Ministry of Education Shanghai Ocean University Shanghai China; ^4^ Fujian Ocean Innovation Center Xiamen China

**Keywords:** allotetraploidization, common carp, population genetics, subspecies divergence, transposable elements

## Abstract

An important question in evolutionary biology is how polyploidization generates raw material for phenotypic diversification. Transposable elements (TEs) represent an underestimated source of genetic variation in eukaryotic genomes. By integrating 516 whole‐genome resequencing datasets and 236 transcriptomes from common carp (*Cyprinus carpio*), a representative allotetraploid fish, we constructed the first population‐scale landscape of TE insertions in teleosts. TE insertions are widespread in the carp genome and preferentially associated with stress‐responsive genes, with DNA transposons as major contributors. Relaxed purifying selection and TE burst events coexist, generating abundant variation for subsequent subspecies differentiation. Compared with a closely related diploid species, carp exhibits more exonic TE insertions and shorter TE–gene distances, and multiple TE superfamilies expanded during tetraploidization. Genome‐wide association analyses uncovered intragenic TE variants underlying domesticated traits missed by SNPs, including DNA transposon deletions associated with scale reduction and altered body shape. Notably, lighter‐colored individuals harbor homozygous deletions of LTR and DNA transposons within *mdfic2*, whose knockout in zebrafish reduces pigmentation. Most trait‐associated variants reflect lineage‐specific loss of ancient TE insertions rather than recent transposition. Overall, these findings highlight the distinct role of TEs in polyploid genome evolution and phenotypic diversification, providing new insights into TE dynamics in vertebrates.

## Graphical abstract

By integrating 516 whole‐genome resequencing datasets and 236 transcriptomes of allotetraploid common carp, this study establishes the first population‐scale atlas of transposable element (TE) variation in teleost species. TE bursts, relaxed purifying selection, and lineage‐specific loss of ancient insertions shape genome evolution and phenotypic diversification, highlighting transposable elements as important drivers of vertebrate evolutionary innovation.

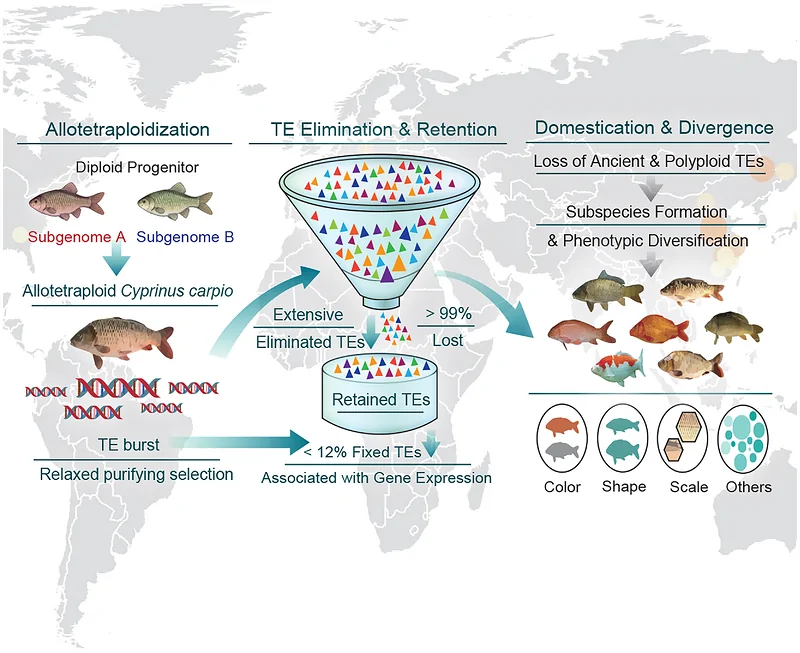

## 1 Introduction

Transposable elements (TEs) are ubiquitous components of eukaryotic genomes and have long been recognized as major drivers of genomic innovation [1, 2]. As important sources of genetic instability, although the majority of TE insertions are efficiently removed by purifying selection to maintain genome integrity, a small proportion can be retained and even evolve into novel functional genes or regulatory elements [3]. Accumulating evidence from both plants and animals demonstrates that TE activity can reshape gene regulatory networks and generate genetic variation with phenotypic consequences [4, 5]. Nevertheless, transposable elements remain an often underestimated source of genetic variation in population genetics. TE insertions can generate mutations with potentially large phenotypic effects by disrupting gene structures, modifying cis/trans‐regulatory elements, or creating new regulatory pathways [6]. In many cases, such structural variants (SVs) have been shown to promote important phenotypic differentiation [7]. At the same time, polyploid genomes provide particularly rich information for investigating TE dynamics and their evolutionary process. Polyploidization is frequently accompanied by increases in TE content, and both TE bursts and relaxed purifying selection may contribute at different stages of whole‐genome duplication events [8, 9]. In plants, extensive hypotheses have been proposed to explain the effects of these two pathways, including the accumulation of TEs driven by relaxed purifying selection in autotetraploid *Arabidopsis thaliana* [10], and the classic “genomic shock” hypothesis related to interspecific hybridization and allopolyploidy [11]. However, similar population‐level studies in polyploid animals remain rare.

Compared with higher vertebrates, fishes (especially cyprinids and salmonids) represent one of the most typical and recurrent examples of polyploidy [12], providing valuable systems for studying how TE dynamics shape genome and phenotypic evolution in vertebrates. In recent years, genomic resources and genetic tools for many fish species have expanded rapidly, resulting in increasingly dense genetic maps and higher‐resolution population analyses [13, 14]. Nevertheless, in contrast to studies in crops, most analyses of the genetic mechanisms for fish domestication traits still primarily focus on genome‐wide selection signals or association analysis of single nucleotide polymorphisms (SNPs) and short indels, although recent pangenome and structural variation studies have increasingly reported TE–SV associations [6, 15]. TE insertions themselves remain relatively underexplored as primary variants. Instead, they are often grouped into broader categories of structural variation or indirectly represented by nearby SNP markers [16], leading to an underestimation of their independent genetic contributions. Common carp (*Cyprinus carpio*) is a powerful model for studying genetic evolution following whole‐genome duplication. As a typical allotetraploid species with two subgenomes (AABB), common carp originated through hybridization and allopolyploidization approximately 1.2 million years ago and exhibits extensive environmental adaptability [17]. Also, as one of the earliest domesticated fish species, artificial breeding of common carp can be traced back to the Neolithic Age, 8000 years ago [18]. Over a long evolutionary history, common carp has adapted to both artificial cultivation and natural environments. During prolonged processes of domestication and geographic expansion, carp populations gradually diverged into multiple evolutionary strains and developed extensive phenotypic variation [19], including skin pigmentation, scale reduction, and body shape diversification. Importantly, compared with many other aquaculture species, common carp is supported by abundant genomic resources, including population‐scale resequencing and regulatory networks [17], providing a unique opportunity to evaluate the contribution of TEs to polyploid genome evolution and animal phenotypic diversity.

Here, we systematically analyzed 516 publicly available whole‐genome resequencing datasets of common carp and constructed a comprehensive population‐scale TE insertion landscape. By combining TE insertions with gene expression data, subspecies differentiation, and TE‐based genome‐wide association studies (TE‐GWAS), we investigated how TE dynamics associated with allopolyploidization and contributed to trait variation in common carp. We demonstrate that TE insertions act as largely independent and functionally relevant genetic variants, and reveal the long‐term impact of ancient TE insertions originating around the period of allotetraploid formation. Combined with experimental evidence, lineage‐specific loss of the ancient insertions plays an essential role in shaping major domestication‐related traits. Overall, our findings highlight transposable elements as a dynamic source of genetic variation that potentially contributes to vertebrate phenotypic evolution and subspecies differentiation.

## 2 Results

### 2.1 TE Polymorphisms as Population Genetic Variations

**FIGURE 1:**
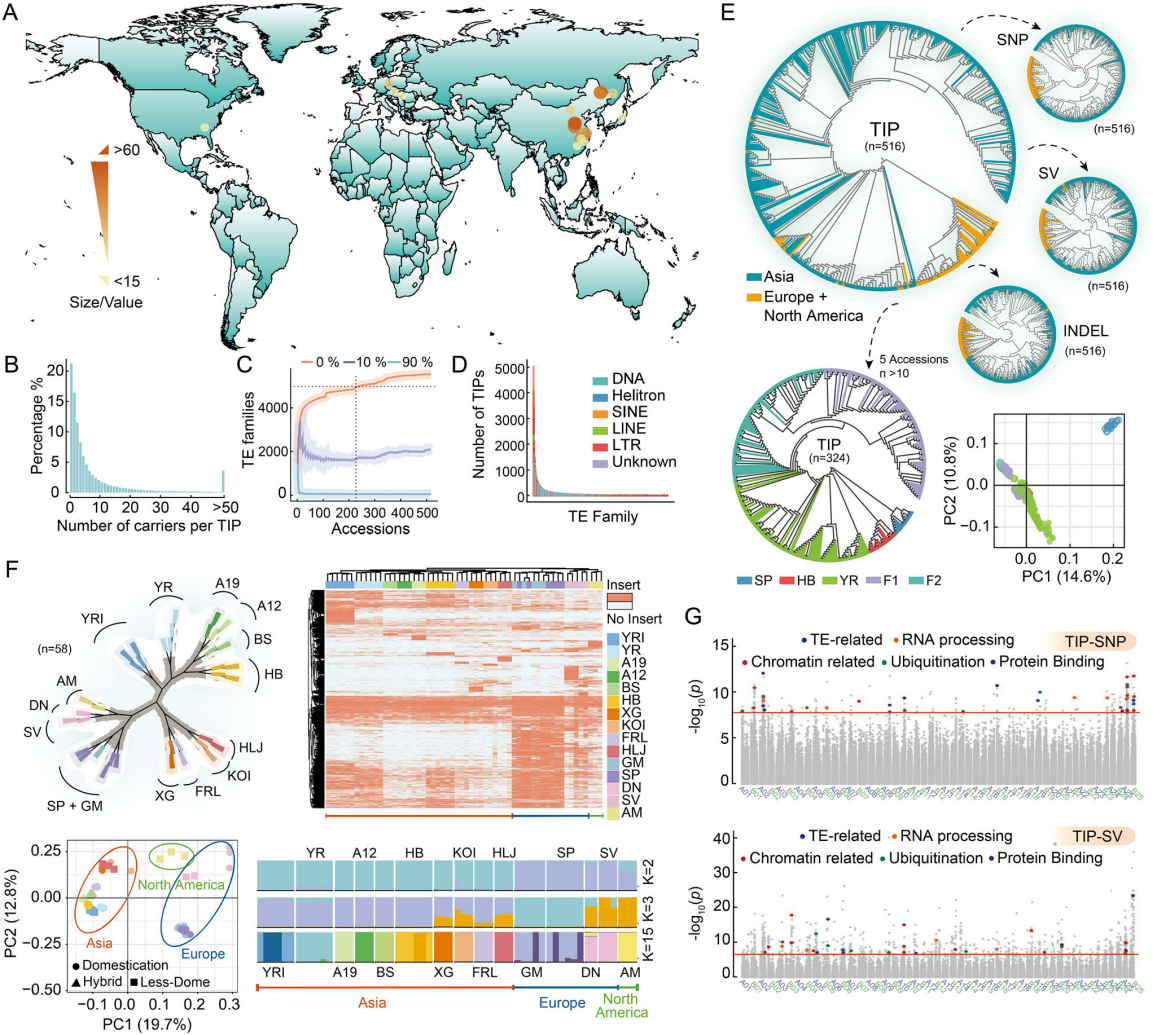
Landscape of TE variation in common carp. A) Geographical distribution of individuals. Global sampling locations of the 516 common carp individuals used in this study. Circle size and color intensity reflect the number of samples per location. B) Frequency distribution of TIP carriers across the population. C) TE family saturation analysis. 0%: Total TE families identified. 10%: Families with insertions present in >10% of individuals. 90%: Families with insertions present in >90% of individuals. The dashed lines indicate that 236 individuals are sufficient to capture 90% of the total TE families. D) Total number of TIPs in each TE family. E) Comparative phylogenomic analysis based on TIPs, SNPs, SVs, and indels. F) Population structure of 15 carp strains. G) GWAS of TE insertion burden. Manhattan plots for the first principal component of the TIP matrix, highlighting host genetic loci associated with chromatin organization and genome regulation.

A total of 3 602 600 transposable elements (TEs) were annotated in the common carp reference genome, including 119 044 intact TEs belonging to 21 superfamilies. To characterize TE insertion polymorphisms at the population level, we analyzed whole‐genome resequencing (WGRS) data from 516 common carp individuals (Figure 1A, Table S1). Using the refined SPLITREADER–TEPID pipeline, we identified 411 597 transposable element insertion polymorphisms (TIPs) across all populations. Most TIPs were present at low frequencies, with 78.86% detected in fewer than 10 individuals, indicating that TE insertions in common carp are largely rare variants (Figure 1B). 90% of the 5739 TE families were observed in fewer than 250 individuals, which was similar to that reported in tomato (Figure 1C) [20]. Overall, the superfamily composition of TE insertion polymorphisms broadly resembled that of reference TEs in the carp genome (Table S2). Similar to the reference TE landscape, TIPs were dominated by Class II DNA transposons, particularly members of the TcMar (25.00%), hAT (11.45%), and PIF‐Harbinger (9.10%) superfamilies. Among Class I retrotransposons, long terminal repeat (LTR) elements were the most prevalent, including Gypsy (14.78%), Retrovirus (2.03%), and DIRS (1.32%) superfamilies (Figure 1D, Table S2).

In addition to TE insertion polymorphisms, we identified 10 931 614 SNPs, 1 403 758 indels, and 430 755 structural variants (SVs) from the same set of 516 WGRS datasets. Phylogenies based on TE insertions, SNPs, indels, and SVs overall consistently differentiated carp populations from Asian and other geographic regions. Phylogenetic reconstruction and principal component analysis (PCA) based on TE insertions clearly separated the five major carp populations, distinguishing four Asian populations (YR, HB, F1, F2) from the European‐introduced SP population, indicating that transposable element insertions have strong potential as population genetic variants (Figure 1E). We further performed a detailed analysis using TE insertions from 15 representative strains. Consistent with established SNP‐based phylogenetic relationships reported in a previous study [21], PCA and genetic structure analysis revealed two major genetic components, with Asian strains grouping together, European strains forming a distinct cluster, and the North American strain positioned intermediate between them (Figure 1F). To explore the genetic basis of TE insertion burden, we conducted genome‐wide association analyses (GWAS) using SNPs and SVs for the first principal component (PC1) from TE insertion polymorphisms (Figure 1G). GWAS based on both SNPs and SVs suggested that host genetic variation contributes to TE insertion patterns. Numerous significantly associated genes were enriched for chromatin accessibility and genome regulation, including transposition activity, RNA processing, chromatin organization, ubiquitination, and protein binding (Figure 1G, Table S3). Collectively, these results support TE insertions as an informative form of population genetic variation.

### 2.2 TE Landscape and Transcriptional Impact in the Carp Genome

**FIGURE 2:**
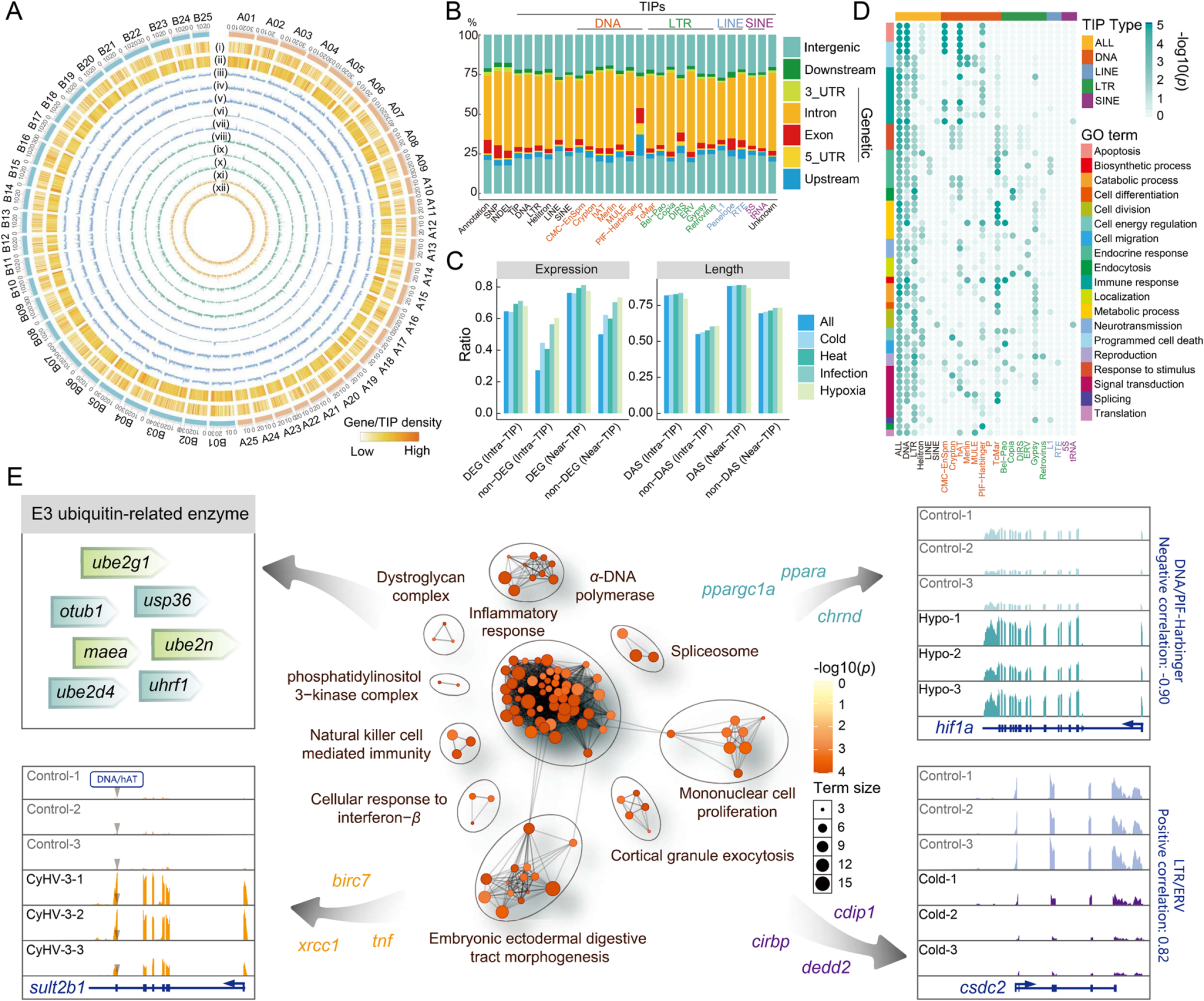
Genome‐wide distribution and potential functional impact of TE insertions. A) The density of genes and TE insertions by superfamily in the common carp reference genome. (i) annotated genes, (ii) TIPs, (iii) TcMar, (iv) hAT, (v) PIF‐Harbinger, (vi) CMC‐EnSpm, (vii) MULE, (viii) Gypsy, (ix) Retrovirus, (x) DIRS, (xi) L1, (xii) Helitron. B) Distribution of TIPs and other genomic variants across gene features. C) Transcriptional effects of TE insertions on nearby genes. The ratio of differentially expressed genes (DEGs) and differentially alternatively spliced (DAS) genes with intra‐genic or near‐genic (within 1 kb) TIPs under temperature stress, infection, and hypoxia. D) GO analysis of genes with exonic TE insertions. E) TE‐gene co‐expression and functional enrichment network.

TE insertions and genes were distributed across the entire common carp reference genome, and both showed a relatively even chromosomal distribution, whereas TIPs exhibited a mild enrichment toward chromosomal ends (Figure 2A). Despite this overall pattern, pronounced differences in insertion preferences were observed among TE superfamilies. TIPs from DNA transposon superfamilies such as TcMar and hAT were broadly distributed across genic and intergenic regions, while CMC‐EnSpm, MULE, and several LTR retrotransposon families showed a stronger tendency to accumulate toward telomeric regions on specific chromosomes (Figure 2A). Compared with other types of genomic variants, TE insertions were obviously reduced in exonic regions, indicating selective constraints against genetic insertions in the carp genome (Figure 2B). Notably, several recently active TE superfamilies [22, 23], including P and DIRS elements, exhibited relatively higher proportions of exonic insertions, potentially reflecting insufficient time for purifying selection to remove deleterious insertions (Figure 2B). To assess the transcriptional impact of TE insertions, we collected 236 RNA‐seq datasets from diverse experimental conditions (Table S4), including pathogen infection [24, 25], temperature stress [26, 27], and hypoxia [28, 29]. Differentially expressed genes (DEGs) or differentially alternatively spliced (DAS) genes were more likely to harbor TE insertions within 1 kb flanking regions of their gene bodies compared with non‐DEGs or non‐DAS genes. Depending on the stress conditions, 64.15–71.04% of DEGs contained TIPs, whereas only 27.21–60.29% of non‐DEGs harbored TIPs. Similarly, 79.52–83.54% of DAS genes were associated with TIPs, compared with 54.94–60.53% of non‐DAS genes (Figure 2C). We further performed Gene Ontology (GO) enrichment analyses of genes with TE insertions into exonic regions. Several DNA transposon superfamilies showed significant enrichment in terms related to environmental stress response. In particular, hAT and CMC‐EnSpm elements were overrepresented in apoptosis and stress‐associated biosynthetic processes, differing from the stress‐responsive activation of Copia elements commonly reported in plants (Figure 2D, Table S5) [30].

To better understand the transcriptional coordination between transposable elements and protein‐coding genes, we identified differentially expressed TEs (DETEs) under multiple conditions with a two‐fold change threshold. Spearman correlation analysis across all individuals revealed that genes co‐expressed with TEs under any condition were significantly enriched in several stress and immunity response terms, with the majority showing positive expression correlations (Figure 2E, Figure S1, Table S6). Many TE‐targeted genes were involved in ubiquitination processes, implying a potential role of TE activity in protein stability regulation. Under specific stress conditions, distinct gene–TE co‐expression patterns were observed. For instance, *sult2b1* encodes a sulfotransferase involved in steroid and xenobiotic metabolism and is associated with immune responses and pathogen defense in teleost fish [31]. In CyHV‐3 viral infection strains, *sult2b1* harbored a DNA/hAT insertion within its fifth exon and displayed strong co‐expression with the corresponding TE. Under cold stress, the cold shock domain‐containing protein gene *cscd2* showed a highly positive correlation with an LTR/ERV transposon. The expression of a hypoxia‐inducible factor gene *hif1a*, was significantly negatively correlated with a DNA/PIF‐Harbinger element under hypoxic conditions. Although only a small fraction of TE families (less than 12%) showed consistent co‐expression with host genes, these retained TEs may regulate gene expression through cis‐/trans‐regulatory interactions, reflecting the dual nature of transposable element insertions as both potentially deleterious and regulatory.

### 2.3 Relaxed Purifying Selection in Polyploid and TE Loss

**FIGURE 3:**
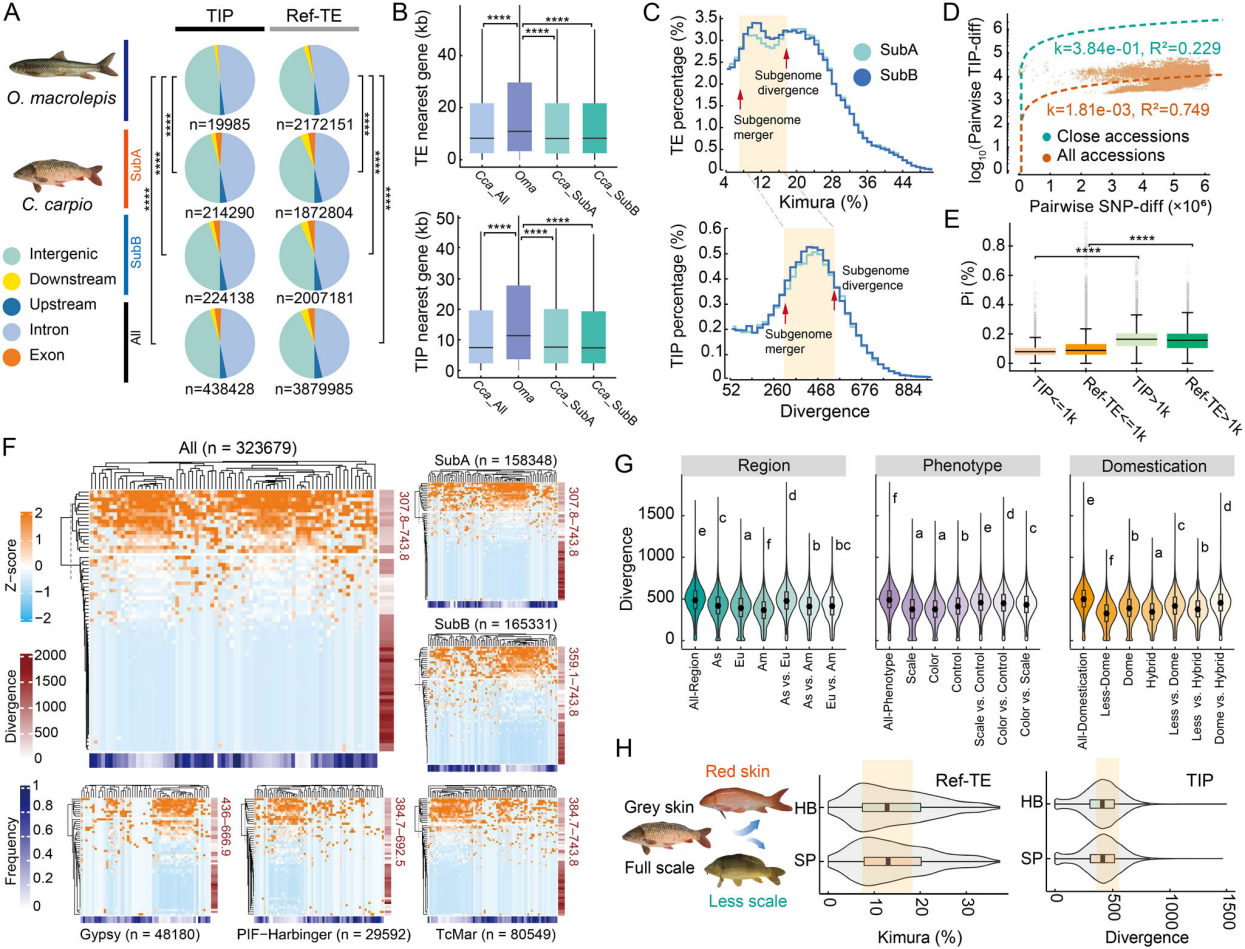
Historical dynamics of TE insertions in common carp. A) Genomic distribution of TE insertions in diploid *Onychostoma macrolepis* and common carp. B) Distance of intergenic TE insertions to the nearest gene. C) Estimation of subgenome divergence and merger phases using Kimura distances (%) for Ref‐TEs and SNP‐based divergence for TIPs. The orange shading represents the evolutionary interval between progenitor divergence and allotetraploid formation. D) Selection‐driven TE removal. Pairwise SNP differences and pairwise TIP differences across all individuals. The actual number of retained insertions is orders of magnitude lower than expected. Blue dashed line: close individuals; Orange dashed line: all individuals. E) SNP nucleotide diversity (π) near TE insertions. ^****^: *p* < 0.0001. F) Age–frequency heatmaps of TE insertions. Heatmaps displaying the abundance (Z‐score), divergence (age), and carrier frequency of TE insertions. The color gradient from blue to orange indicates an increasing Z‐score of TE insertion abundance. G) TE insertion age distributions for different carp populations, including geographical distribution, phenotypic traits, and domestication history. Boxes represent the interquartile range. In the Region category, AS, EU, and AM represent Asia‐specific, Europe‐specific, and America‐specific TE insertions, respectively; AS vs. EU, AS vs. AM, and EU vs. AM represent TE insertions shared between the corresponding two geographic groups but absent from the third group; All‐Region represents TE insertions shared among all three geographic groups. The same classification strategy was applied to Phenotype and Domestication categories, where “vs.” indicates shared TE insertions between two groups and “All” indicates TE insertions shared among all groups. H) Selective TE retention during subspecies differentiation and phenotypic variation. YR: Yellow River carp, grey skin, full scale. HB: Hebao carp, red skin. SP: Songpu carp, reduced scale. The orange shading represents the evolutionary interval between progenitor divergence and allotetraploid formation. Boxes represent the interquartile range.

Polyploidization is usually accompanied by relaxed purifying selection against transposable element insertions [32]. Previous studies based on one or several common carp and related cyprinid genomes have been used to explore TE dynamics, including subgenome evolution and expansion of specific TE families [33, 34, 35]. To further evaluate whether similar patterns also apply to allotetraploid common carp at the population level, we additionally performed TE insertion polymorphism detection using WGRS data from 56 individuals of *Onychostoma macrolepis*, a closely related diploid species. TE insertions within exonic regions in *O. macrolepis* were significantly reduced compared to the whole genome and two subgenomes of common carp, approximately 10% of the exonic insertion content detected in common carp (Figure 3A). Both TIPs and reference TEs in *O. macrolepis* genome were located at significantly greater distances from the nearest genes compared with those in common carp (Figure 3B). Together, these patterns indicated stronger purifying selection against TE insertions in the diploid lineage and suggested that allotetraploid common carp has experienced relatively relaxed purifying selection following polyploidization. To further investigate the evolutionary dynamics of TE insertions, we focused on TIPs shared by at least two genomes and estimated their relative ages by quantifying the number of SNPs accumulated within 50 kb upstream and downstream of each insertion site, as described in *Arabidopsis thaliana* [36]. The TE landscape based on TIP divergence showed evolutionary transition windows similar to those obtained using Kimura values. Specifically, both identified an early separation between the two ancestral carp subgenomes and a subsequent fusion associated with allotetraploidization (Figure 3C). The polyploidization‐related transition identified from Kimura divergence (7–18%) was also recovered using TIP divergence estimates (312–546), further supporting the ability of population‐level TE landscapes to capture major evolutionary signals. The regression between two relative temporal frameworks revealed a consistent trend across TE families (R^2^ = 0.87) (Figure S2A), providing additional support for the use of TIP divergence to characterize TE evolutionary dynamics.

We further evaluated the overall extent of transposable element removal based on closely related individuals (individuals with the lowest 5% of genome‐wide SNP divergence). The most divergent individuals differed by ∼50 000 TIPs. If TIPs across all individuals accumulated at the same rate as closely related individuals, the number of retained insertions would be two to three orders of magnitude higher than observed (Figure 3D). However, the actual insertion number was much lower than expected, suggesting a large number of TE insertions effectively cleared during evolution. We estimated that approximately 99.8% of TE insertions in common carp may have been eliminated by purifying selection or related processes (Figure 3D). The estimated proportion of TE insertions substantially exceeds that of missense mutations (45.83%) and nonsense mutations (58.63%) (Figure S2). Consistent with strong selective constraint, nucleotide diversity (π) of SNPs within 1 kb of TE insertions was significantly lower than that in other regions (Figure 3E). This evidence consistently supports the strong repulsion of transposable insertions at the genome level. Although the tetraploidization may contribute to relaxed purifying selection, TEs remain subject to strong long‐term negative selection, limiting their persistence in the carp genome.

### 2.4 TE Bursts During Polyploidization and Subsequent Differential Retention

Consistent with the hypothesis that allopolyploidization is often accompanied by short‐term bursts of activity in TE families, age–frequency heatmaps based on TE insertions revealed a significant enrichment of TE activity during and before the tetraploidization phase of common carp (Figure 3F). During the evolutionary interval between progenitor divergence and allotetraploid formation, both subgenome A and subgenome B showed TE insertion hotspots, and most TE superfamilies displayed evidence of transient activity bursts. Particularly, strong signals were observed for Class I Gypsy retrotransposons and Class II DNA transposons, including TcMar, PIF‐Harbinger, and hAT, suggesting the potential occurrence of transposon bursts existed during allopolyploidization in the common carp genome (Figure 3F, Figure S3).

To explore whether TE insertions were differentially retained or eliminated during subsequent evolutionary and domestication processes, we compared the age distributions of TE insertions across geographic groups, major phenotypes, and domestication status (Figure 3G). For geographic differentiation, TE insertions unique to populations in a single continent showed significantly lower divergence ages than those shared between regions or across all populations. This pattern is consistent with inheritance from a more recent common ancestor of geographically proximate populations. Among region‐specific insertions, divergence ages followed a clear gradient (Asia > Europe > America), whereas insertions shared between Asia and Europe showed higher divergence ages than those shared between Asia and America or Europe and America. From Asia to Europe and subsequently to North America, the retention of ancient TEs continued to decline, accompanied by the emergence of region‐specific novel TE insertions (Figure 3G, Figure S4A). These patterns are consistent with the domestication and migration history of ancestral groups retaining more ancient TE insertions, with common carp initially domesticated in East Asia, introduced westward into Europe during the Roman period, and subsequently brought to North America from Europe in the late nineteenth century [18, 37]. Similarly, for phenotypic differentiation, TE insertions shared across all populations exhibited significantly older divergence ages than those specific to single phenotypic groups. Groups with reduced scales and pigmented skin showed significantly younger ages relative to grey and fully scaled groups (Figure 3G, Figure S4B). These phenotypes are known to have emerged relatively late during domestication through a combination of rare mutational events and strong artificial selection, whereas the grey body color with full scales represents a more ancestral state [38]. Accordingly, the population characteristics of TE insertions were broadly consistent with the fixation history of specific phenotypes in common carp. For domestication intensity, from insertions shared across all populations to those fixed in domesticated groups, then to recently hybridized populations, and less domesticated populations, the divergence ages of TE insertions significantly decreased (Figure 3G, Figure S4C). This suggested that long‐term artificial selection and inbreeding facilitate the fixation of a subset of TE insertions in domesticated lineages. In contrast, hybridization introduces heterogeneous, lineage‐specific insertions which are unlikely to reach complete fixation over short time scales, whereas less domesticated populations retained a higher proportion of insertions with low frequency and individual specificity. To identify which ages of TE insertions are primarily associated with phenotypic differences, we compared patterns of TE changes in three representative strains, including HB (red skin), SP (reduced scales), and YR (grey skin with full scales). Interestingly, 46.95–54.62% of the lost TEs in HB and SP were inserted during tetraploidization (Figure 3H). The selective retention or elimination of these ancient insertions may contribute to subspecies differentiation and key phenotypic emergence in common carp.

### 2.5 TEs as an Important Source for Phenotypic Variation in Common Carp

**FIGURE 4:**
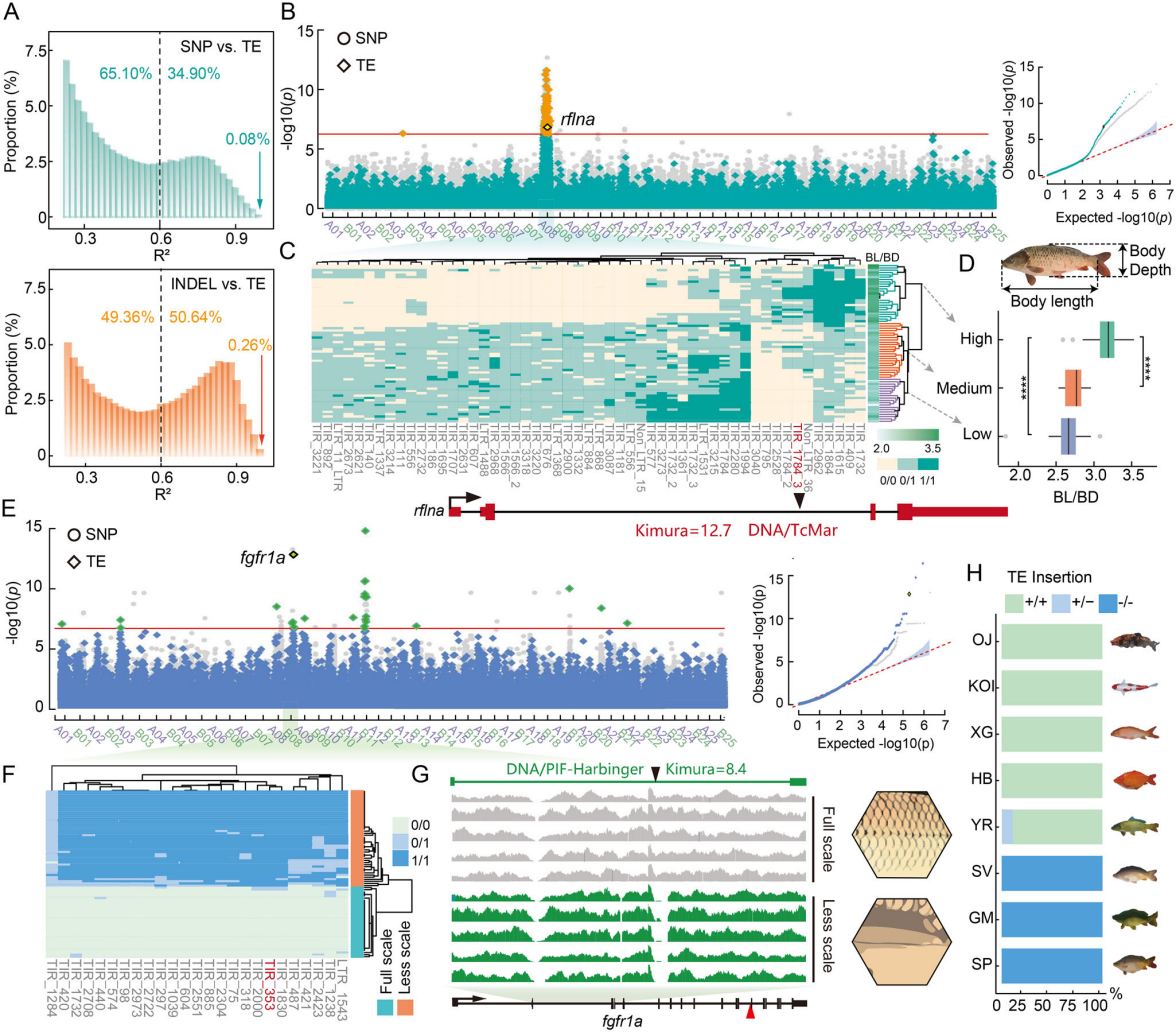
TE‐GWAS for key phenotypic traits in common carp. A) Linkage disequilibrium (LD) between TEs and SNPs/indels within 1 kb flanking windows. B) TE‐GWAS for body shape. BL/BD: the ratio of body length to body depth. Grey circles: SNP markers. Diamonds: TE insertions. Red line: significance threshold. C) Genotyping heatmap of significant TE variations associated with body shape. D) Comparison of BL/BD based on heatmap clustering. Boxes represent the interquartile range. ^****^: *p* < 0.0001. E) TE‐GWAS for scale pattern. Grey circles: SNP markers. Diamonds: TE insertions. Red line: significance threshold. F) Genotyping heatmap of TE variations for scale patterns. G) A homozygous TE deletion within *fgfr1a* in scale‐reduced individuals. Relative positions of the DNA/PIF‐Harbinger TE polymorphism identified in this study (black triangle) and the previously reported structural deletion (red triangle) within *fgfr1a*. H) Population‐level TE insertion frequency. The bar chart shows the percentage of TE genotypes (+/+, +/‐, ‐/‐) across representative strains. Scale‐reduced strains (SV, GM, and SP) consistently exhibit the homozygous TE absence (‐/‐) within the candidate gene.

To systematically evaluate whether TEs represent an important source of phenotypic evolution in common carp, we first assessed the linkage disequilibrium (LD) between TE insertions and nearby SNPs and indels within a 1 kb flanking window. Even when restricting the analysis to flanking variants showing moderate linkage with TE insertions (*r* ^2^ > 0.2), the proportion of TE–SNP (0.08%) and TE–indel (0.26%) pairs approaching complete linkage was substantially lower than that of SNP–SNP (2.5%), SNP–indel (4.0%), or indel–indel (14.9%) pairs (Figure 4A, Figure S5). This pattern indicates that, even when located near other variants, TE insertions still tend to capture genetic variation that is largely independent from nearby SNPs and indels, suggesting that they may represent an additional and underexplored source of genetic diversity. Given this relative independence between TE insertions and nearby small variants, we reasoned that TE‐based association analyses might reveal genetic signals not readily captured by SNP‐based GWAS. Based on this possibility, we performed TE‐based genome‐wide association studies (TE‐GWAS) on two major phenotypic traits for common carp, including body shape (a quantitative trait) and scale pattern (a qualitative trait). Previous SNP‐based GWAS and selection signals identified candidate genomic regions for both traits, within which *rflna* and *fgfr1a* were proposed as candidate genes [35, 39, 40]. Our analyses further revealed TE variants within these candidate genes, providing validation of our TE‐GWAS method.

For body shape measured as the ratio of body length to body depth (BL/BD), the structural variation observed in *rflna* actually corresponds to a trace of DNA transposon deletion (Figure 4B, Table S7). The genotyping heatmap of significant TE variants clustered individuals into three distinct groups, one with significantly higher BL/BD values. A DNA/TcMar TE variant was identified within the second intron of *rflna*. Individuals with higher BL/BD ratios were more likely to exhibit a heterozygous or homozygous absence of this TE insertion, whereas the individuals with the lowest ratios largely retained clearer ancestral insertions (Figure 4C,D). PCR genotyping further showed that individuals carrying DNA/TcMar deletions exhibited significantly higher BL/BD ratios than insertion carriers (*p* < 0.0001), consistent with trends identified by population‐scale TE‐GWAS (Figure S6A,B). Similarly, TE‐GWAS for scale pattern identified a homozygous deletion of a DNA transposon within *fgfr1a* (Figure 4E, Table S7). Compared with the quantitative body shape trait, the significant TE phenotyping of the qualitative scale trait was clearly divided into different phenotypes. Nearly all scale‐reduced individuals carried homozygous TE deletions, while fully scaled individuals retained ancestral insertions (Figure 4F). Although an SV associated with the scale reduction has previously been identified at *fgfr1a*, with scale‐reduced individuals carrying the homozygous deletion [35, 40], we found a distinct DNA/PIF‐Harbinger variant located within intron 2 of *fgfr1a* (Figure 4G). This DNA/PIF‐Harbinger transposon showed homozygous absence specifically in scale‐reduced individuals. Population‐level analysis further supported this association, as scaleless or scale‐reduced strains (SP, GM, and SV) consistently lacked the TE insertion in the homozygous state, in contrast to other strains (Figure 4H). PCR genotyping confirmed the population‐scale TE genotyping results, with the DNA/PIF‐Harbinger deletion detected only in the scaleless SP strain and absent from the scaled YR and HB strains (Figure S6C). In addition, QQ plots indicated that TE‐GWAS signals for both traits were stronger than SNP‐based GWAS. The estimated ages of the TE insertions within the candidate genes of both traits predate the tetraploidization, which supported their subsequent loss may contribute to the emergence of key phenotypic traits.

### 2.6 An Unregistered TE Variation for Carp Body Color

**FIGURE 5:**
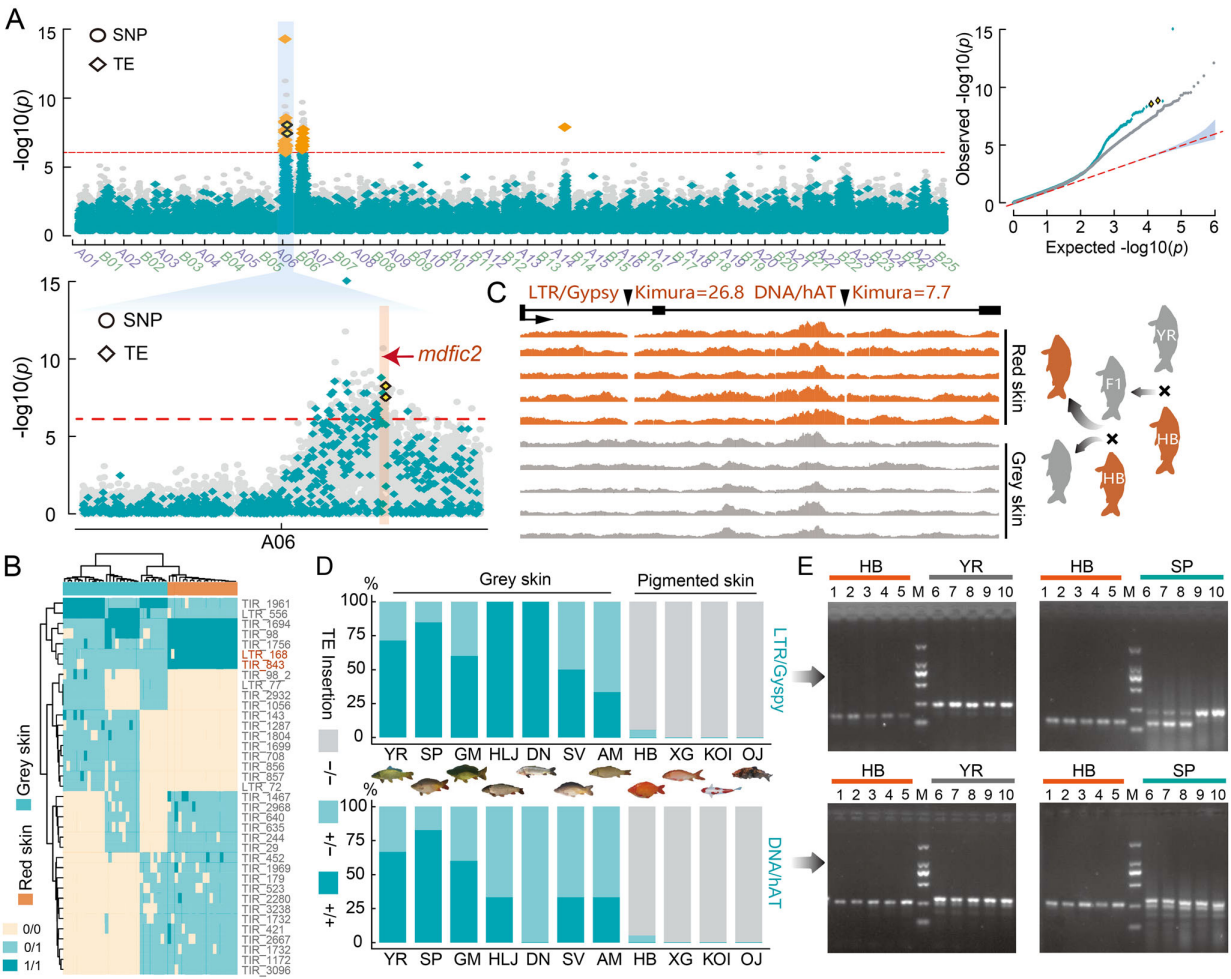
TE deletions are associated with carp body color. A) TE‐GWAS for body color. Grey circles: SNP markers. Diamonds: TE insertions. Red line: significance threshold. B) Genotyping heatmap of significant TE variations for carp body color. C) Two homozygous TE deletions within *mdfic2* in red‐skinned individuals. D) Population‐level TE insertion frequency for two TE variations. Bar charts show the frequency of TE genotypes across diverse strains. Pigmented skin strains (HB, XG, KOI, OJ) are consistently homozygous for the absence (‐/‐) of both TE insertions. E) PCR validation for TE deletions associated with body color. Gel electrophoresis images confirm the stable homozygous absence of one LTR/Gypsy insertion and one DNA/hAT insertion in the HB carp strain. Lane M represents the DNA marker, and from bottom to top: 100, 250, 500, 750, 1000, and 2000 bp. Target band sizes are consistent with the predicted absence of TE insertions in pigmented individuals. The uncropped pictures are listed in Figure S6.

We further performed TE‐GWAS for carp body color using an F2 backcross family derived from YR carp (grey skin) and HB carp (red skin) [41]. The most significant TE signals were detected on the homologous chromosomes A06 and B06, with 19 genes on A06 and 8 genes on B06 harboring intragenic TE variants (Table S8). Notably, 88% of candidate genes exhibited asymmetric TE variation between the two subgenomes, with TE variants detected only in one homologous copy, indicating divergent TE contributions between the two subgenomes. We focused on significant traces of TE activity within an unexplored gene, *mdfic2*, located on chromosome A06 (Figure 5A, Table S8). Specifically, a 101 bp deletion in the first intron and a 43 bp deletion in the second intron were detected as part of an LTR/Gypsy transposon and a DNA/hAT transposon, respectively. Both deletions were homozygous in red‐skinned individuals, while grey‐skinned individuals were heterozygous or completely retained the TE insertions (Figure 5B,C). The two candidate TE variants were in strong linkage disequilibrium in the backcross population (*r* ^2^ = 0.888), indicating that they largely co‐segregate during inheritance. After extending the analysis to broader strains, we found individuals with pigmented skin were consistently homozygous for the absence of two TE insertions, while grey skin strains were either heterozygous or wild‐type phenotype (Figure 5D). PCR‐based genotyping of representative YR, HB, and SP strains further validated the stability of these homozygous deletions in pigmented skin individuals (Figure 5E, Figure S6). To assess population differentiation at these loci, we compared TE insertion frequencies and fixation index (*F_ST_*) values among YR, HB, and SP strains (Figure S5, Table S9). The top 1% TE‐F_ST_ loci mapped to *mdfic2* (body color, *F_ST_* = 0.68), *rflna* (body shape, *F_ST_* = 0.51), and *fgfr1a* (scale pattern, *F_ST_* = 0.59), supporting the sensitivity of TE to potential genetic variation and major phenotypes.

**FIGURE 6:**
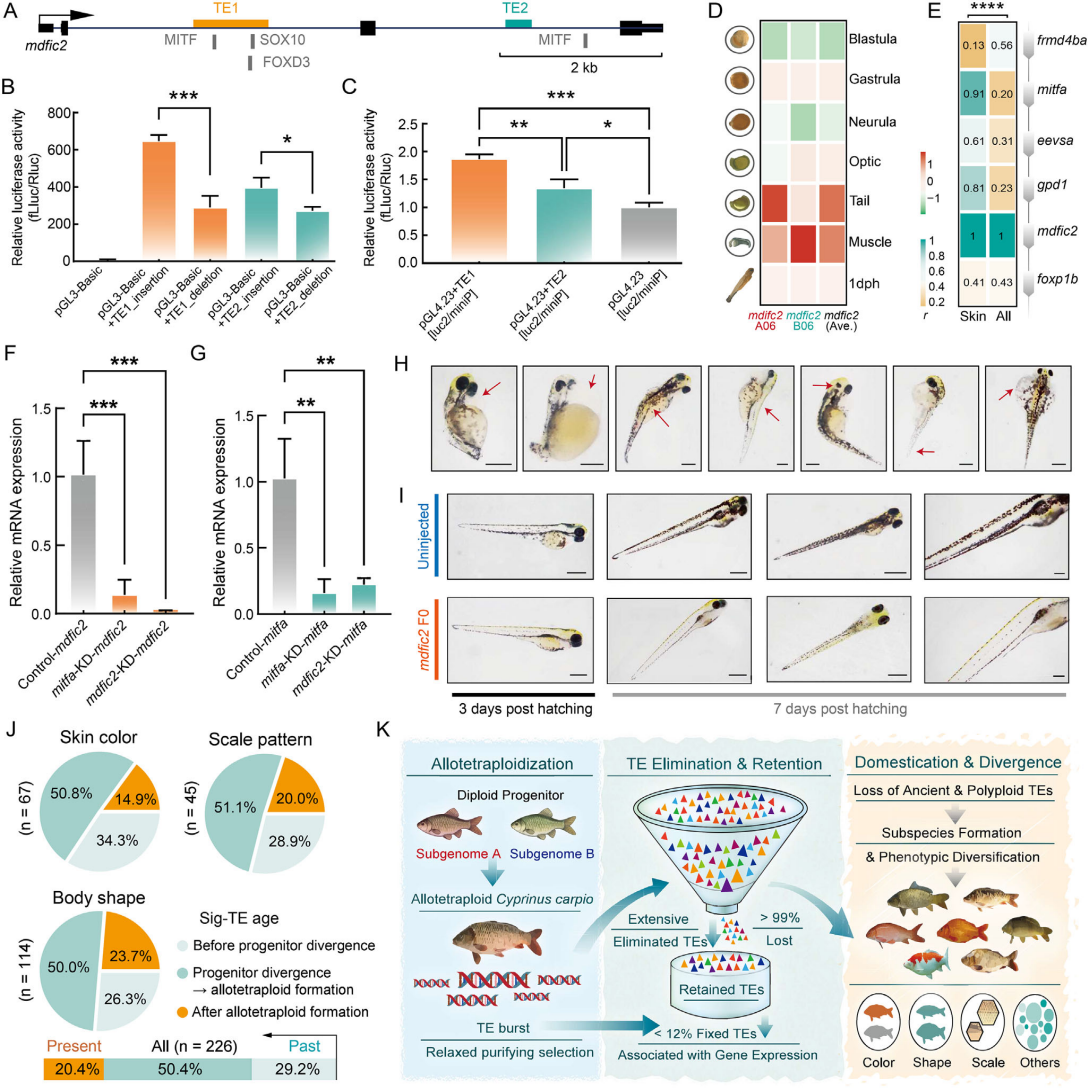
Functional validation of TE‐mediated regulation of *mdfic2* associated with carp pigmentation. A) Predicted transcription factor binding sites (TFBS) for pigmentation regulators (MITF, SOX10, and FOXD3). TE1: LTR/Gypsy transposon. TE2: DNA/hAT transposon. B) Allele‐specific dual‐luciferase reporter assays evaluating the regulatory effects of TE insertion and deletion alleles. Genomic fragments containing either the TE insertion or deletion allele were cloned into the pGL3‐Basic reporter vector, and transcriptional activity was compared between alternative TE alleles. ^*^: *p* < 0.05. ^***^: *p* < 0.001. C) Enhancer activity assays of candidate TE sequences using the pGL4.23 minimal promoter reporter system. Both TE1 and TE2 sequences exhibit enhancer activity, with TE1 showing stronger transcriptional activation than TE2. ^*^: *p* < 0.05. ^**^: *p* < 0.01. ^***^: *p* < 0.001. D) Embryonic expression of *mdfic2* in YR carp strain. Heatmap shows high expression of *mdfic2* during late embryonic development, particularly at the tail bud and muscle effector stages, coinciding with pigment cell development. E) Skin‐specific co‐expression of *mdfic2*. Correlation heatmap of *mdfic2* and nearby genes within a conserved gene cluster. *mdfic2* exhibits strong co‐expression with *mitfa* specifically in skin tissue. ^****^: *p* < 0.0001. F) Relative expression of *mdfic2* in the injection control, *mitfa* knockdown, and *mdfic2* knockdown groups, as determined by qRT‐PCR. *mdfic2* expression was reduced following both *mitfa* and *mdfic2* knockdown. ^***^: *p* < 0.001. G) Relative expression of *mitfa* in the injection control, *mitfa* knockdown, and *mdfic2* knockdown groups, as determined by qRT‐PCR. *mitfa* expression was significantly reduced following *mitfa* knockdown and was also reduced following *mdfic2* knockdown. ^**^: *p* < 0.01. H) Morphological defects in zebrafish *mdfic2* mutants. Representative phenotypes of F0 mutants generated via CRISPR–Cas9‐mediated knockout. Developmental abnormalities include (from left to right): pericardial edema, ocular defects (microphthalmia), spinal curvature, and shortened body length. Red arrows indicate specific pathological sites. Scale bars: 500 µm. I) Reduced melanin pigmentation in *mdfic2* mutants. Comparison between uninjected controls and *mdfic2* F0 mutants at 3 and 7 days post‐hatching (dph). Mutants exhibit a significant reduction in body melanin pigmentation compared to the clear pigmentation patterns in control larvae. Scale bars: 500 µm. J) Insertion time distribution of significant TE variants in TE‐GWAS analysis. Pie charts illustrate the estimated ages of TE insertions significantly associated with body color, scale pattern, and body shape. The majority of these variants were inserted before the allotetraploid formation (29.2% + 50.4%) of common carp. K) Evolutionary model of TE‐mediated genomic and phenotypic evolution.

*mdfic2* encodes a MyoD family inhibitory protein which might position upstream in neural crest pathways [42]. While functional studies of *mdfic2* remain limited, transcription factor binding site (TFBS) prediction revealed potential binding motifs for pigmentation‐related regulators, including MITF, SOX10, and FOXD3, in proximity to the two intronic TE variants (Figure 6A). To directly evaluate whether these intronic TE variants possess regulatory activity, we performed allele‐specific dual‐luciferase reporter assays comparing the TE insertion and deletion alleles. For both candidate loci, the insertion alleles exhibited higher transcriptional activity than the deletion alleles. The TE1 (LTR/Gypsy) insertion allele increased reporter activity by approximately 2.2‐fold compared with the deletion allele, whereas the TE2 (DNA/hAT) insertion allele showed an approximately 1.45‐fold increase (Figure 6B). Furthermore, when the TE sequences were cloned upstream of a minimal promoter, both TE1 and TE2 enhanced reporter expression, with TE1 displaying stronger enhancer activity than TE2 (Figure 6C). These results indicate that the two intronic TE sequences possess intrinsic cis‐regulatory activity and that their deletion may alter the regulatory potential related to *mdfic2*. In the common carp genome, *mdfic2* was retained in both subgenomes and showed high expression during late embryonic stages, including eye vesicle, tail bud, and muscle contraction stages, coinciding with the onset of pigment cell development (Figure 6D). Meanwhile, *mdfic2* showed skin‐specific co‐expression with several nearby genes, particularly *mitfa*, a pattern not observed in other tissues (Figure 6E). This locus resides within a conserved teleost gene cluster (*foxp1b*–*mdfic2*–*mt‐ox*–*eevsa*–*mitfa*–*frmd4ba*), in which *mitfa*, *eevsa*, and *mt‐ox* have been experimentally validated to play roles in pigmentation [43, 44, 45, 46]. In common carp, the knockdown of *mdfic2* was accompanied by a significant reduction in *mitfa* expression (Figure 6F). Similarly, the knockdown of *mitfa* was also accompanied by a significant decrease in *mdfic2* expression (Figure 6G). These expression changes are consistent with the strong co‐expression relationship observed between the two genes and suggest that *mdfic2* may participate in the *mitfa*‐associated pigmentation regulatory pathway, potentially contributing to the regulation of body pigmentation in common carp.

To further explore the functional relevance of *mdfic2*, we performed CRISPR–Cas9–mediated knockout in zebrafish, which harbors a single‐copy ortholog of this gene. F0 mutants were successfully generated, while most individuals exhibited severe developmental abnormalities, including pericardial edema, ocular defects, skeletal malformations, and reduced viability (Fig 6H, Figure S7), suggesting that *mdfic2* is essential for normal development. Nevertheless, among surviving F0 larvae observed within seven days post‐hatch, approximately 20% (11/56) displayed reduced body melanin pigmentation rather than complete loss, indicating the potential role of *mdfic2* in pigment regulation (Figure 6I). Finally, we summarized the estimated ages of all significant TE variants identified in the TE‐GWAS analyses. The majority of these TEs were fixed in the carp genome either during the evolutionary interval between progenitor divergence and allotetraploid formation (50.4%) or before progenitor divergence (29.2%) (Figure 6J), followed by lineage‐specific loss during domestication, coinciding with phenotypic diversification and subspecies formation in common carp.

## 3 Discussion

As one of the earliest fish species subjected to systematic genetic and genomic analyses, common carp has accumulated extensive genomic resources in recent years [17, 47]. On the one hand, common carp is a representative allotetraploid teleost and an excellent model for studying ploidy evolution in fishes under whole‐genome duplication [34]. Continuous improvements in carp genome assemblies and comparative genomic analyses have advanced our understanding of allopolyploid formation and historical dynamics between the two subgenomes. On the other hand, compared with many diploid fish, common carp shows stronger environmental adaptability [48], and understanding its adaptive evolution requires integrative analyses incorporating transcriptomic and other functional genomic data. In addition, as one of the earliest domesticated fish species, common carp displays a wide range of phenotypic variation and has been widely cultivated worldwide [49, 50]. The discrimination of various strains and the development of breeding tools rely heavily on whole‐genome sequencing analyses at multiple population scales. In this study, transposable elements serve as a bridge linking polyploid genome evolution to phenotypic diversification analysis. By integrating the latest Yellow River carp reference genome with 516 whole‐genome resequencing datasets, we were able to characterize non‐reference TE insertions besides those present in the carp genome. This comprehensive TE landscape revealed that TE insertions in the common carp genome are non‐random but preferentially associated with stress‐related genes and pathways, with DNA transposons playing a dominant role. Unlike the Copia elements frequently highlighted in plants [51]. DNA transposons belonging to hAT and CMC‐EnSpm superfamilies appear to be the primary contributors to environmental response in common carp. This difference also reflects lineage‐specific TE landscapes, as DNA transposons always play prominent roles in teleost genome evolution. For example, Tc1/mariner elements represent one of the major active DNA transposon groups in several teleost genomes, including salmonid and six neoteleost species [52, 53].

At the same time, TE insertions represent a class of genomic variation that can provide temporal information and offer insights into evolutionary events in the common carp genome. Polyploidization is generally associated with relaxed purifying selection due to gene redundancy, which could reduce the efficiency of eliminating deleterious mutations, as observed in the over‐accumulation of transposable elements in autotetraploid *Arabidopsis arenosa* [10], Vertebrate polyploid lineages can also experience TE accumulation following genome duplication. In rainbow trout, the ancestral whole‐genome duplication was followed by extensive rediploidization and restructuring of duplicated genomic regions after whole‐genome duplication [54]. Another common hypothesis in polyploidy evolution is that allopolyploidization is accompanied by a short burst of activity in several TE families [55, 56]. TE dynamics have been increasingly recognized as an important component of genome evolution in common carp. With the availability of high‐quality reference genomes, Xu et al. have used TE sequence divergence to investigate the divergence and subsequent merger of ancestral subgenomes [34]. Such analyses relied on the divergence accumulated within reference TEs and interpreted TE abundance patterns across divergence intervals to reconstruct major evolutionary transitions. Our study provides a complementary population‐based perspective by estimating relative divergence of population TIPs from flanking sequence variation. Although reference TEs and TIPs could represent different levels of genomic contexts, both reflect sequence changes accumulated after TE insertion events. TIP divergence is not intended as a direct conversion of Kimura divergence into absolute time, but rather as an independent relative temporal framework for examining TE evolutionary dynamics. The recovery of similar subgenome divergence and fusion patterns from population TE landscapes supports the biological relevance of this approach.

In addition, specific TE families, such as TcMar and LINE elements, have been suggested to experience expansion or burst‐like activity accompanied by allopolyploidization [33]. Similar to these genome‐level analyses, our population‐scale TIP divergence captured comparable evolutionary signatures of ancestral subgenome dynamics and revealed periods of elevated TE activity during the polyploidization process. By exploring population TE dynamics during allopolyploid evolution, we found that genome‐wide TE bursts could coexist with relaxed purifying selection, thereby accumulating raw genetic material for phenotypic diversification of common carp. Because TE retention reflects a balance between transposition, selection, and evolutionary time, estimating long‐term TE elimination requires comparisons among individuals spanning sufficient genetic divergence. Recently derived populations or closely related breeding lineages may contain insufficient evolutionary resolution to capture cumulative TE turnover dynamics. These historical dynamics do not conflict with the long‐term purging of TEs in the common carp genome, as the vast majority of transposable elements are continuously removed to maintain genomic stability. Among the few retained TE insertions, only a small fraction evolves into regulatory elements participating in gene expression or contributes to the generation of subspecies (Figure 6K). However, TE evolutionary dynamics in polyploid genomes could also be shaped by factors such as subgenome‐specific evolutionary trajectories and lineage‐specific TE loss. Although insertion frequency is often considered a simplified indicator of TE age, this relationship is not deterministic because TE persistence depends on the balance between transposition activity, selection, and population history [57, 58]. Our comparison between TIP divergence and insertion frequency further supports the need for caution when using frequency alone to infer TE evolutionary history.

It is well established that transposable elements usually experience strong genome‐wide purging while simultaneously representing a major source of structural variation [59, 60]. In our phylogenetic analyses, although TIP, SNP, indel, and SV show overall consistent population differentiation, minor topological discordance was observed among variant classes. Such discordance may arise because these variant classes capture partially overlapping but non‐identical aspects of evolutionary history. In particular, TE insertions are shaped by lineage/family‐specific transposition activity, insertion age, allele frequency, and selection, with many polymorphic insertions occurring at low frequency and some TE variants showing limited linkage with nearby SNPs [16, 57, 61, 62]. SVs and indels also encompass heterogeneous mutation types with distinct mutational mechanisms and evolutionary dynamics [63, 64]. Thus, the TE‐based phylogeny may capture evolutionary signals that are not fully represented by SNPs, indels, or SVs, rather than simply representing a different reconstruction of population history. The occasional clustering of European individuals with Asian populations in the TE‐based tree may further reflect the retention of shared or ancestral TE polymorphisms following the relatively recent introduction of European carp. However, the direct contribution of TE insertions themselves as genetic variants remains underappreciated. TEs might contribute largely independent genetic signals because of the extensive weak linkage disequilibrium between TE variants and surrounding SNPs/indels. Compared with classic SNP‐based GWAS, TE‐based association signals can in some cases be stronger and more directly localized within candidate genes, such as the DNA transposon variants associated with body shape and scale pattern in common carp. Such low‐frequency genetic signals may reveal additional sources of genetic contribution not captured by SNPs, such as the homozygous loss of TE insertions at *mdfic2* in pigmented skin individuals. The allele‐specific reporter assays further support that TE polymorphisms within the *mdfic2* locus may contribute to regulatory variation associated with pigmentation.

Interestingly, significant TE variation showed a strong subgenome‐specific pattern between homologous chromosomes. Similar asymmetric accumulation of structural variation between duplicated subgenomes has been observed in polyploid genomes [65, 66]. The *mdfic2* copy on A06 harbored two pigmentation‐associated TE deletions, whereas no significant TE/SNP variants were detected in the B06 copy. However, the absence of detectable variants does not necessarily indicate that the B06 copy retains an ancestral function, as homologous genes could still undergo functional divergence through regulatory changes or expression differences. Similar subgenome‐specific TE patterns were also observed for other genes, such as A06‐specific *ror1*, *mast2*, and *gabrb3*, and B06‐specific *slc9a7* and *cadps*. The contributions of duplicated genes to pigmentation regulation are complex. Although most candidates showed asymmetric variation, several genes contained variants in both homologous copies. For example, *col7a1* and *lrp8* carried TE variants in both A06 and B06 copies, while *oca2*, a previous candidate gene involved in melanosome function and melanin production [67], harbored SNP variants in both subgenomes. Nevertheless, whether these duplicated copies function equivalently or asymmetrically remains to be determined through future expression and functional exploration. In addition, a comprehensive exploration of genetic variation related to carp traits may benefit from the combination of approaches using TE variants, SNPs, indels, SVs, and other forms of variation. For instance, the canonical pigmentation regulator *mitfa* could not be directly identified by TE‐GWAS, and our manual inspection found that a previously reported color‐associated structural variant at this locus may not be TE‐derived [41]. Similarly, at the *fgfr1a* locus, a previously reported ∼300 bp structural deletion in the same gene was not captured by TE‐GWAS [35, 40], because it could not be represented as an annotated TE insertion. TE‐GWAS specifically captures TE‐derived structural variation rather than all forms of genomic variants. Across multiple traits, the associated TE variants consistently trace back to the formation of the allotetraploid genome or earlier. Obviously, subspecies differentiation in common carp is tightly coupled with phenotypic divergence, motivating us to evaluate the overall contribution of TE activity to this process. Even when restricting the analysis to TE variants showing significant differentiation among phenotypic groups, approximately half of these insertions originated between progenitor divergence and allotetraploid formation, approaching 80% if the proportion before subgenomic segregation is included. In fact, TE differentiation in pigmented skin, reduced‐scale, and less‐elongated body shape groups is dominated by the loss or gain of ancient TE insertions rather than by the accumulation of novel TE insertions.

Compared with well‐studied crop systems such as rice [68], rapeseed [69], and tomato [20], the exploration of transposable elements in aquatic animals remains in its early stages. Split‐read and discordant‐read approaches provide a practical framework for extending TE insertion analyses to large populations, but their resolution depends on both read support and the genomic context of each insertion. Highly repetitive flanking sequences or repetitive inserted elements can produce ambiguous read mapping and obscure insertion boundaries [70], while short‐read SV approaches similarly have reduced resolution for repetitive regions and complex breakpoint structures [71, 72]. We systematically evaluated the determinants of variant recovery in our dataset (Figure S8), which indicate that coverage might primarily affect whether sufficient split‐/discordant‐read evidence is available for individual TIPs, while the recoverability of SVs and TIPs could also be influenced by the local sequence architecture. Meanwhile, the allotetraploid architecture of common carp provides an additional genomic context. Although similar analytical frameworks have been applied in other polyploids [10, 73], homologous regions can still introduce multiple related sequences into a single reference genome. When sequence divergence between homeologs is insufficient, ambiguous or failed read mapping occurs [74], which may either underestimate true insertions or falsely assign them to the wrong subgenome. Therefore, Future population‐scale long‐read sequencing, haplotype‐resolved and pangenomic assemblies will be valuable for extending the present landscape to TE insertions and SVs within highly repetitive or closely related homologous regions, and for determining how much additional TE‐mediated variation remains unresolved in the duplicated genomes. Although artificial selection and demographic bottlenecks might promote the fixation or removal of functionally relevant TE variants, whether similar patterns of TE differentiation are more evident in other domesticated or selectively bred carp strains, such as those selected for rapid growth or disease resistance, remains to be investigated.

In summary, combining rich allotetraploid carp genomic, resequencing, and transcriptomic resources, we constructed the first comprehensive TE insertion landscape in teleosts. TE insertions are widespread in the common carp genome and preferentially cluster near stress‐responsive genes, particularly various DNA transposons. Compared with closely related diploids, relaxed purifying selection and TE bursts during the formation of the allotetraploid genome have accumulated abundant TE variation in the common carp genome, providing raw material for phenotypic diversity. As underappreciated genetic variants, TE insertions could reveal effects undetectable by other genetic variations. Strain‐specific TE variants localize to candidate genes for several carp traits, including body shape (*rflna*), scale pattern (*fgfr1a*), and body color (*mdfic2*). Ancient TE insertions during and prior to allotetraploidization were partially lost during domestication and subspecies divergence, highlighting their contribution to phenotypic differentiation in common carp. These findings underscore TEs as both historical records of genome evolution and as functional contributors to adaptive and domesticated traits.

## 4 Experimental Section

### 4.1 Data Collection

Detailed information on the data used in this study was listed in Table S1 and Table S4. In total, whole‐genome resequencing (WGRS) data of 516 *Cyprinus carpio* individuals and 56 *Onychostoma macrolepis* individuals were collected for further genotyping. 236 paired‐end RNA‐seq libraries from different conditions were collected for expression analysis. All datasets were obtained from the National Center for Biotechnology Information (NCBI) and the China National Center for Bioinformation (CNCB) [75].

### 4.2 Identification of TE Insertion Polymorphisms (TIPs) in C. Carpio

A combination of SPLITREADER and TEPID pipeline [76] was used to detect and filter TIPs in *C. carpio*. Briefly, all raw WGRS reads were first mapped to the *C. carpio* reference genome by BWA‐MEM (v 0.7.17‐r1188) [77], and sorted using SAMtools (v1.9.2) [78]. The sorted files were filtered and removed duplicated reads with Picard (v3.0.0) [79]. Reference TEs of *C. carpio* were identified de novo from the reference genome using HiTE (v3.3.3) [80], and only intact TEs were retained as a TE library for SPLITREADER (v1.3) and TEPID (v0.10). Putative insertions were further filtered based on positive coverage and negative coverage drop with default parameters, generating a TIP presence/absence matrix. Specifically, insertions were encoded as 1 for samples in which the insertion passed the filtering criteria, 0 for samples in which the insertion was absent and the reference site was sufficiently covered (>5 reads), and NA for samples in which the reference coverage was insufficient to confidently determine the absence of the insertion. To further reduce potential false‐negative and false‐positive assignments, for each candidate TIP, insertion‐supporting reads were required to be consistent with the expected local insertion structure, and the target‐site duplication (TSD) characteristics expected for each TE family were considered when evaluating candidate insertion sites. TIP loci for which no individual was assigned as an insertion carrier (1), with all remaining states coded as 0 or NA, were excluded from the final TIP matrix. Density of reference TEs and TIPs for the top 1% superfamilies displayed in the circos plot was calculated in 100‐kb windows by BEDTools (v2.31.1) [81].

### 4.3 Validation of TIPs

To empirically assess the reliability of TE insertion variants, 200 randomly selected TIP loci were manually inspected using IGV (v2.18.4) [82], across 25 individuals representing five major population groups (YR, SP, HB, F1, and F2). The individuals included 10 samples with ∼6× sequencing depth and 15 samples with ∼15× sequencing depth (200 × 25 = 5000 independent evaluations). Overall, manual inspection supported a precision of ∼ 83% (FDR: ∼17%) and a sensitivity of ∼76% (FNR: ∼24%) (Figure S8). The validation rate increased from ∼77% at ∼6× sequencing depth to ∼86% at ∼15×, whereas the false‐negative rate decreased from approximately 32% to 20%, respectively. Furthermore, we examined the relationship between depth and the number of detected TE families and TIPs across the 516 individuals. The numbers of TE families and TIPs showed positive relationships with sequencing depth (R^2^ = 0.266 and 0.268, respectively). To further assess the specificity of population‐level TE variants, we independently evaluated TIPs using chromosome‐level genome assemblies from two additional *C. carpio* strains, HB and SP (CNCB: PRJNA510861, PRJCA002174). TIPs were transferred to the HB and SP assemblies, and the corresponding genomic regions were compared with independently annotated reference TE sequences in each assembly. Approximately 83% of TIPs in the HB genome and 76% of TIPs in the SP genome overlapped an independently annotated reference TE within ±1 kb, which were similar to those reported in other TIP detection methods based on short reads [20, 76].

Two candidate TE deletions identified by TE‐based GWAS for skin color were further validated by polymerase chain reaction (PCR) using genomic DNA extracted from 25 *C. carpio* individuals (10 YR carp, 10 HB carp, and 5 SP carp). For the scale‐associated TE insertion, PCR validation was performed using 24 individuals from three representative strains (SP, scaleless, n = 8; YR, fully scaled, n = 8; HB, fully scaled, n = 8). For the body shape‐associated TE locus, 24 independent individuals (12 deletion carriers and 12 insertion carriers) were genotyped by PCR, and the body length/body depth (BL/BD) ratio was compared between genotypic groups. The primers for these TE variations were designed using the NCBI Primer‐BLAST tool (https://www.ncbi.nlm.nih.gov/tools/primer‐blast/) [83] and detailed information on reagents and reaction conditions is provided in Table S11. The presence or absence of TE deletions was determined based on the expected fragment size differences between individuals.

### 4.4 Variant Calling and Population Genetic Analyses

Other genomic variants, including single‐nucleotide polymorphisms (SNPs), insertions/deletions (indels), and structural variants (SVs), were also identified from aligned WGRS data. SNPs and indels were called using the NVIDIA Clara Parabricks (v4.4.0) GPU‐accelerated pipeline with default parameters, generating raw gVCF files for each individual [84]. All gVCF files were merged using GATK (v4.2.5.0) CombineGVCFs [85], followed by joint genotyping with GATK GenotypeGVCFs. Variant quality control for SNPs and indels was performed using GATK VariantFiltration and PLINK (v1.90) to remove variants with a minor allele frequency (MAF) < 0.1 [86]. Structural variants were detected using the “Germline SV calling” model of DELLY (v1.3.3) [87]. SV calling was first performed for each individual to generate individual BCF files. SV sites were then merged into a unified site list, and genotyping was conducted across all samples. Genotyped SVs from all individuals were subsequently merged to produce a final SV VCF file using BCFtools (v1.14) [78]. We examined the relationship between depth and the number of detected SVs across the 516 individuals. The number of SVs showed a weak positive relationship with sequencing depth (R^2^ = 0.014) (Figure S8C). The YR reference genome was aligned against the HB and SP chromosome‐level assemblies using Minimap2 (v2.28‐r1209) [88], and assembly‐based structural differences were identified using SyRI (v1.8.2) [89]. These SVs supported by assemblies were subsequently compared with the population‐level SVs after coordinate conversion. Across 516 samples, ∼89% and ∼81% of population‐level SV calls were independently supported by the HB and SP assemblies (Figure S8E), respectively, which provides independent evidence that the majority of population‐level SV calls are supported by chromosome‐level genome assemblies. As the TIP detection pipeline described above only generated a presence/absence matrix, we additionally organized a genotyping file for reference TEs containing only TE loci overlapped with structural variant regions.

Following the method described in tomato [20], phylogenetic trees based on transposon insertions were created from the final binary matrix using the parallelDist package and ape package in R (v4.4.1) [90]. SNP/indel/SV‐based phylogenetic trees were constructed under the nucleotide substitution model using FastTree (v 2.1.11) [91]. All trees were visualized using iTOL (https://itol.embl.de) [92]. Principal component analysis (PCA) was performed using the PLINK PCA module, and population genetic structure was inferred using ADMIXTURE (v1.3.0) [93].

### 4.5 Expression Analysis of TEs and Protein‐Coding Genes

Clean RNA‐seq reads were first aligned to *C. carpio* reference genome using STAR (2.7.11b) [94]. For assembly and expression quantification, the software TEtranscript (v2.2.3), specially designed for both transposable elements and protein‐coding genes, was used to identify differentially expressed genes (DEGs) and differentially expressed transposable elements (DETEs) with *p*‐adjust < 0.05 and |log_2_(fold change)| > 2 [95]. Reference TE annotations for TEtranscript software were manually organized. For alternative splicing (AS) analysis, rMATS (v4.2.0) classified AS events as skipped exon [96], alternative 3’ splice site, alternative 5’ splice site, mutually exclusive exon, and retained intron. Differential alternative splicing (DAS) genes under different conditions were defined based on a false discovery rate (FDR) < 0.05. Gene Ontology (GO) functional annotation for *C. carpio* was generated using eggNOG‐mapper (http://eggnog‐mapper.embl.de/) [97], and GO terms with *p* < 0.05 were considered statistically significant. Spearman correlation coefficients (r) were calculated from the expression matrix between each pair of protein‐coding genes and transposable elements. Potential TE‐target genes (|*r*| > 0.9, *p* < 0.001) were visualized as functional enrichment networks using the R package aPEAR [98]. Transcription factor binding sites (TFBSs) within TE‐targeted genes were predicted based on JASPAR CORE vertebrate database of the UCSC Genome Browser (https://genome.ucsc.edu/) with a minimum score of more than 400 [99].

### 4.6 TE Insertion Time Estimation

Two approaches were employed to estimate the relative ages of TE insertions. For reference TEs, the TE library generated by HiTE (v3.3.3) was provided to RepeatMasker (v4.1.5) to calculate Kimura substitution levels for each TE family as reference TE insertion age [100]. For TIPs, relative insertion ages were estimated following the method reported in *Arabidopsis thaliana* [36]. SNP pairwise divergence was calculated within 100‐kb windows flanking each TIP between all individuals using PLINK (v1.90). The relative age of each TIP was determined based on the maximum pairwise divergence among all individual pairs. To compare the relative temporal information obtained from reference TEs and TIPs, the average Kimura value and the average TIP divergence age for each TE family were calculated. The relationship between these two relative measures was evaluated using a linear regression model without intercept, considering that both values represent accumulated divergence following TE insertions. Meanwhile, whole‐genome SNPs were annotated using SnpEff (v5.4) [101]. To investigate the potential TE clearance rate, pairwise divergence was calculated for both SNPs and TIPs between all pairs of individuals. Individual pairs within the lowest 5% of genome‐wide SNP divergence were considered closely related individuals, for which long‐term purifying selection was expected to have had minimal impact. Linear regression models without intercept were then fitted to examine the relationship between TIP divergence and different categories of SNP divergence, including synonymous, missense, and stop‐gained variants.

### 4.7 Comparative Analysis of Repetitive Elements

For one closely related species, *O. macrolepis*, 56 whole‐genome resequencing (WGRS) datasets were used to identify TIPs and reference TEs following the same pipeline applied to *C. carpio*. 56 whole‐genome resequencing (WGRS) datasets were mapped to the published *O. macrolepis* reference genome (CNCB: PRJCA002622), and the *O. macrolepis* TE library was constructed de novo from this assembly using HiTE. TIPs and reference TEs for *O. macrolepis* were identified separately from those of *C. carpio*, just following the same SPLITREADER–TEPID pipeline. All comparative statistics between the two species were performed after independent TE identification based on each respective genome. To examine potential differences in selection pressure between diploid and tetraploid genomes, the genomic distributions of TE insertions across different genomic regions were calculated using BEDTools (v2.31.1). For TIPs and reference TEs located in intergenic regions, the distance to the nearest annotated genes was calculated and compared between *C. carpio* and *O. macrolepis*. In addition, the nucleotide diversity (π) was calculated and compared for SNPs within 1 kb regions of TE insertions and for regions beyond 1 kb from TE insertion sites using VCFtools (v 0.1.16) [102]. Heatmaps of TIP divergence and TIP frequency across 516 *C. carpio* samples for each TE superfamily were visualized using the R package Pheatmap [103].

### 4.8 TE‐GWAS for Quantitative and Qualitative Traits

For each transposon insertion, linkage disequilibrium (LD) was calculated between the insertion and surrounding SNPs and indels within a 1 kb window using PLINK (v1.90). Only TE–variant pairs with *r* ^2^ > 0.2 were retained and visualized in R (v4.4.1). Phenotype information for three significant domestication‐related traits in *C. carpio*, including body shape, body color, and scale pattern associated with subspecies differentiation, was listed in Table S10. Genome‐wide association studies (GWAS) based on both SNPs and TE variants were performed for these three traits using a mixed linear model (MLM) in GEMMA (v0.98.3) [104]. For the quantitative trait body shape, the ratio of body length to body depth measured in F1 hybrid populations from Yellow River (YR) carp and Hebao (HB) carp was used as the phenotypic value. For the two qualitative traits, body color and scale pattern, skin color (red vs. grey) and scale pattern (reduced vs. normal) were encoded as binary phenotypes. The Wald test was applied to assess statistical significance, and significance thresholds were defined using Bonferroni‐corrected *p*‐values. Manhattan and quantile–quantile (QQ) plots were generated using the CMplot package in R (v4.4.1).

We further focused on TE insertions that were both statistically significant in GWAS and located within annotated gene regions. Genotyping heatmaps of these TE variants were visualized using the pheatmap package in R (v4.4.1), and candidate genes and associated TE insertions were inspected using IGV (v2.18.4) [82]. Additionally, TE‐based population differentiation (F_ST_) was calculated for the two qualitative traits. Only the top 1% of TE‐F_ST_ values were retained as candidate divergent TE loci. Thresholds were set at TE‐F_ST_ > 0.406 for body color (YR vs. HB) and TE‐F_ST_ > 0.570 for scale pattern (YR vs. SP). Genotyping frequencies of candidate TE loci were summarized using PLINK –hardy.

### 4.9 Dual‐Luciferase Reporter Assay

(i) To evaluate the regulatory activity of the two intronic TE variants within *mdfic2*, dual‐luciferase reporter assays were performed using HEK293T cells. Genomic fragments containing the TE insertion and deletion alleles were synthesized and cloned into the pGL3‐Basic luciferase reporter vector. The resulting constructs, including pGL3‐Basic‐TE1 insertion, pGL3‐Basic‐TE1 deletion, pGL3‐Basic‐TE2 insertion, and pGL3‐Basic‐TE2 deletion, were used to compare the regulatory activity associated with the alternative TE alleles. (ii) To evaluate the intrinsic enhancer activity of the TE sequences, TE1 and TE2 fragments were individually cloned into the pGL4.23 [luc2/minP] reporter vector, which contains a minimal promoter. The empty pGL4.23 [luc2/minP] vector was used as the negative control. HEK293T cells obtained from the Cell Bank of the Chinese Academy of Sciences were cultured in DMEM supplemented with 10% fetal bovine serum. Cells were transfected with reporter plasmids together with the Renilla luciferase control plasmid pRL‐TK using Lipofectamine 3000 (Thermo Fisher Scientific, USA). For the pGL3‐Basic assay, five groups were analyzed: empty pGL3‐Basic vector, TE1 insertion allele, TE1 deletion allele, TE2 insertion allele, and TE2 deletion allele. For the enhancer activity assay, three groups were analyzed: empty pGL4.23 [luc2/minP] vector, pGL4.23‐TE1, and pGL4.23‐TE2. TransDetect Double‐Luciferase Reporter Assay Kit (TransGen Biotech, China) was used to measure Firefly and Renilla luciferase activities, and relative luciferase activity was calculated as the ratio of Firefly luciferase activity to Renilla luciferase activity. Statistical significance was evaluated using one‐way ANOVA with three replicates for each construct. The detailed sequences of all TE insertion/deletion constructs, TE fragments used for reporter assays, and cloning primers are provided in Table S11.

### 4.10 siRNA‐Mediated Knockdown

siRNA‐mediated knockdown (KD) experiments were performed in Yellow River carp (YR) strain. Three siRNAs targeting *mitfa* and three siRNAs targeting *mdfic2* were designed and synthesized by Sangon Biotech Co., Ltd. (Shanghai, China). A non‐targeting siRNA was used as the negative control. The final siRNA sequences were chemically modified with 2′‐O‐methyl, phosphorothioate, and cholesterol modifications to improve their stability in vivo. Based on preliminary screening, the *mitfa*‐261 (KD efficiency: 83.7%) and *mdfic2*‐12 (KD efficiency: 96.3%) were selected for subsequent experiments because of their relatively high knockdown efficiencies. For in vivo delivery, the synthesized siRNAs were complexed with Entranster‐in vivo (Engreen Biosystem, China) at a ratio of 2 µg siRNA to 1 µL transfection reagent and diluted with 10% glucose solution. For fish weighing approximately 30 g, 3 µg siRNA was complexed with 1.5 µL Entranster‐in vivo and administered in a final volume of 30 µL per fish. The complexes were administered once every 48 h for three consecutive injections. Six fish were included in each experimental group, with three biological replicates.

TRIzol (Invitrogen, China) reagent was used to extract total RNA from the collected skin tissues, and reverse transcription was carried out with the Hifair III First Strand cDNA Synthesis Kit (gDNA Digester Plus, Yeasen, China) following the manufacturer's instructions. Then, quantitative real‐time PCR (qRT‐PCR) was performed with the Taq Pro Universal SYBR qPCR Master Mix (Vazyme, China) on a CFX384 Real‐Time PCR Detection System (Bio‐Rad, USA) with three replicates for each 10 µL reaction set. The thermal cycling protocol began at 95°C for 30 s, then proceeded through 40 cycles of 95°C for 10 s and 60°C for 40 s. The gene *β‐actin* served as the reference, and relative expression levels were determined using the 2^−ΔΔCt^ method [105]. All siRNA sequences and qRT‐PCR primer sequences are listed in Table S11.

### 4.11 Gene Editing and Phenotypic Observation

The candidate gene *mdfic2* of TE‐GWAS for carp body color was functionally validated in *D. rerio* (zebrafish). Gene knockout was performed following the method described by Jiang et al. [39] CHOPCHOP online tool (https://chopchop.cbu.uib.no) was used to design single‐guide RNAs (sgRNAs) [106], and the target site was in the third exon of zebrafish *mdfic2*. Forward primers containing the T7 promoter and target sequences (excluding the PAM site) were paired with a universal reverse primer to generate templates for in vitro transcription. The double‐stranded sgRNA templates were then transcribed in vitro using the HiScribe T7 Quick High Yield RNA Synthesis Kit (NEB, #E2040) under RNase‐free conditions, and purified with the Monarch RNA Cleanup Kit (NEB, #T2040L). Validation primers for zebrafish were also designed using NCBI Primer‐BLAST (https://www.ncbi.nlm.nih.gov/tools/primer‐blast/) and synthesized by Sangon Biotech Co., Ltd. (Shanghai, China).

Adult wild‐type zebrafish were maintained in a recirculating aquaculture system under a 14‐h light/10‐h dark photoperiod at 28.0°C, and embryos were obtained from natural spawning. Purified sgRNA (25–35 pg/nL) was mixed with Cas9 protein (250–300 pg/nL) at a 1:1 volume ratio, and 1–2 nL of the mixture was microinjected into one‐cell‐stage wild‐type zebrafish embryos. Control embryos were injected with an equal volume of DEPC‐treated water. PCR amplification of targeted sequences was performed using genomic DNA extracted from F0 embryos and larvae. Sanger sequencing (Shanghai Sangon Biotechnology Co., Ltd., China) was used to confirm the presence of genome editing events. A Nikon ECLIPSE Ni‐U microscope (Nikon, Japan) was used to visually examine phenotypic changes in zebrafish. A Canon EOS 7D digital camera (Canon, Japan) equipped with a Canon EF 100 mm macro lens was then used to capture whole‐body images of juvenile fish. At the end of the experiment (7 days post‐fertilization), larvae were euthanized by overdose of MS‐222 (0.03%). All animal treatments and procedures were approved by the Institutional Animal Care and Use Committee (IACUC) of Xiamen University (Approval No. XMULAC20250427).

## Author Contributions

**Shuimu Hu**: conceptualization, methodology, data curation, investigation, validation, formal analysis, visualization, Writing – original draft, software. **Zhou Jiang**: methodology, investigation, validation, writing – review and editing. **Lin Chen**: writing – review and editing, data curation, supervision, funding acquisition. **Gowena Ling Yi Goh**: writing – review and editing, validation, investigation. **Weijing Li**: writing – review and editing, software. **Qian He**: writing – review and editing, software. **Xintong Chen**: writing – review and editing, software. **Rui Li**: validation. **Yuxuan Liu**: validation. **Ning Li**: writing – review and editing, supervision. **Tao Zhou**: writing – review and editing, supervision. **Peng Xu**: writing – review and editing, conceptualization, project administration, resources, funding acquisition, supervision.

## Funding

The National Science Fund for Distinguished Young Scholars (32225049), National Key R&D Program of China (2019YFE0119000), Postdoctoral Fellowship Program of CPSF (GZB20230386).

## Conflicts of Interest

The authors declare no conflicts of interest.

## Supporting information




**Supporting File 1**: advs77790‐sup‐0001‐SuppMat.pdf.

**Supporting File 2**: advs77790‐sup‐0002‐SuppMat.xlsx.

## Data Availability

Detailed information of whole genome resequencing (WGRS) data of 516 Cyprinus carpio individuals and 56 Onychostoma macrolepis individuals were listed in Table S1. 236 paired‐end RNA‐seq libraries from different conditions were listed in Table S4. All datasets were collected from the National Center for Biotechnology Information (NCBI) and the China National Center for Bioinformation (CNCB) [49].
