# Supplementary material for: Transposable Element Dynamics Drive the Genomic Evolution and Phenotypic Diversification of Allotetraploid Common Carp
Source: Adv Sci (Weinh). 2026 Sep 16:e77790. Online ahead of print. doi: 10.1002/advs.77790 (PMC13583293; doi:10.1002/advs.77790)
Supplement: Supplementary file 1 [file ADVS-9999-e77790-s002.pdf]

Supporting Information

Transposable element dynamics drive the genomic evolution and phenotypic diversification of allotetraploid common carp

Shuimu Hu, Zhou Jiang, Lin Chen, Gowena Ling Yi Goh, Weijing Li, Qian He, Xintong Chen, Rui Li, Yuxuan Liu, Ning Li, Tao Zhou, Peng Xu*

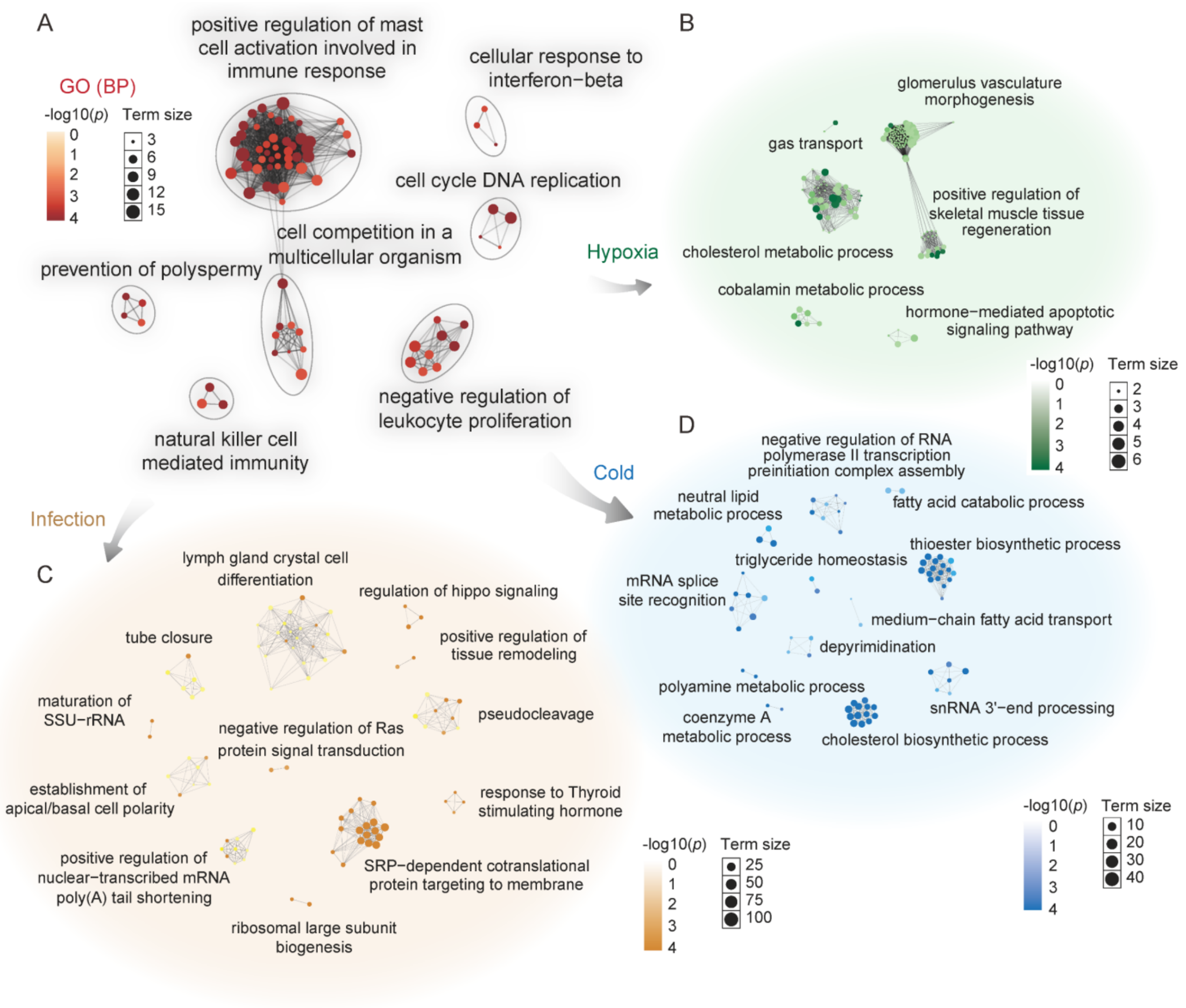

Figure S1. GO biological processes (BP) for TE targeted genes under different conditions, including A) all samples, B) hypoxia, C) infection, D) cold stress.

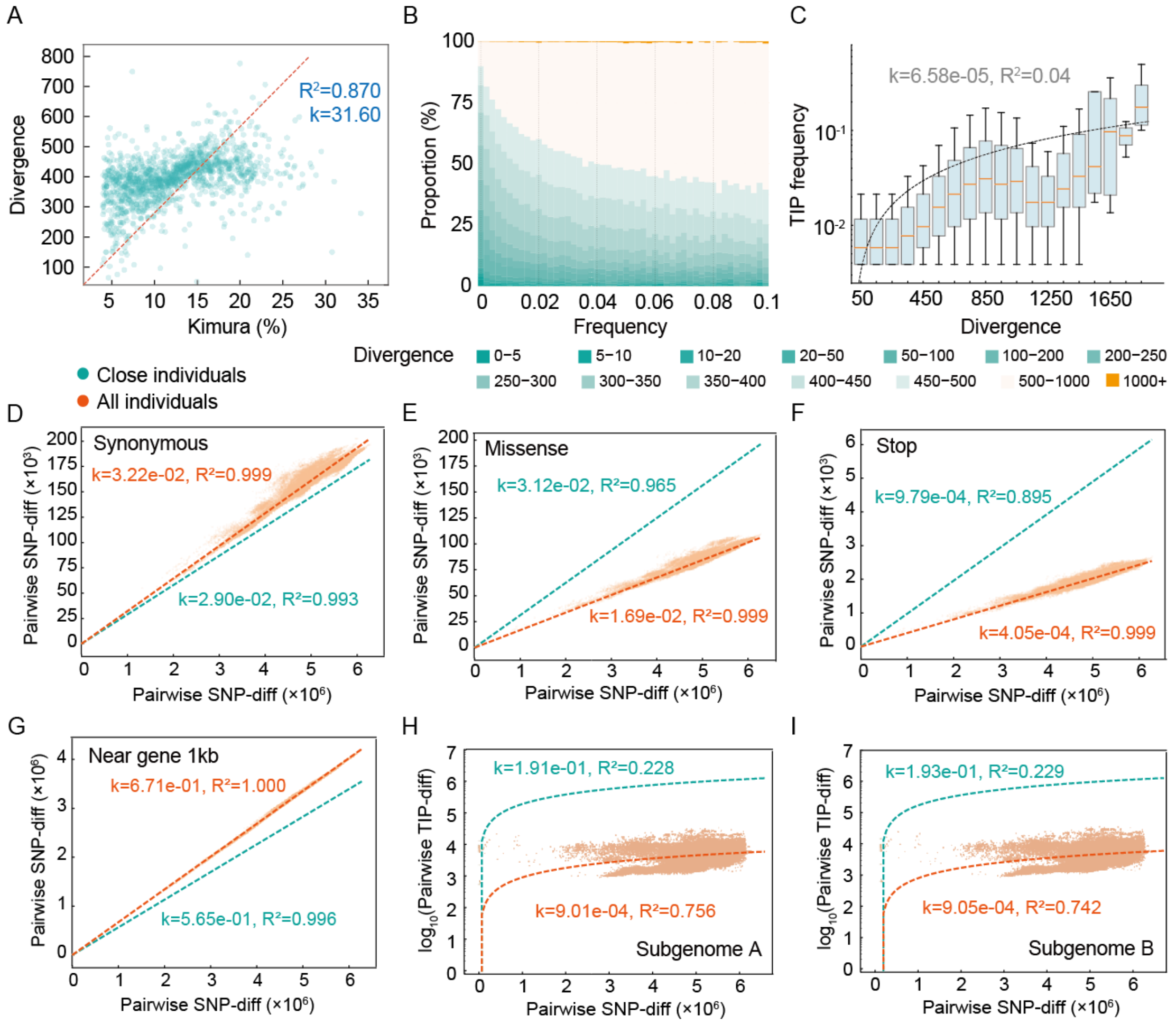

Figure S2. Evolutionary dynamics of TE insertions. A) Comparison between Kimura and TIP divergence estimated ages across TE families. Each point represents one TE family, with the x-axis indicating the average Kimura value of the family and the y-axis indicating the corresponding average TIP divergence age. B) Proportions of TIPs across different allele frequencies, categorized by divergence ages. C) Correlation between TIP frequency and divergence age. C-F) Pairwise SNP-diff correlations in functional regions. Correlations of pairwise SNP differences for (D) synonymous, (E) missense, (F) stop, and (G) near-gene (within 1kb of coding genes) mutations. (H) Pairwise TIP differences in subgenome A. (I) Pairwise TIP differences in subgenome B. Blue dashed line: close individuals; Orange dashed line: all individuals.

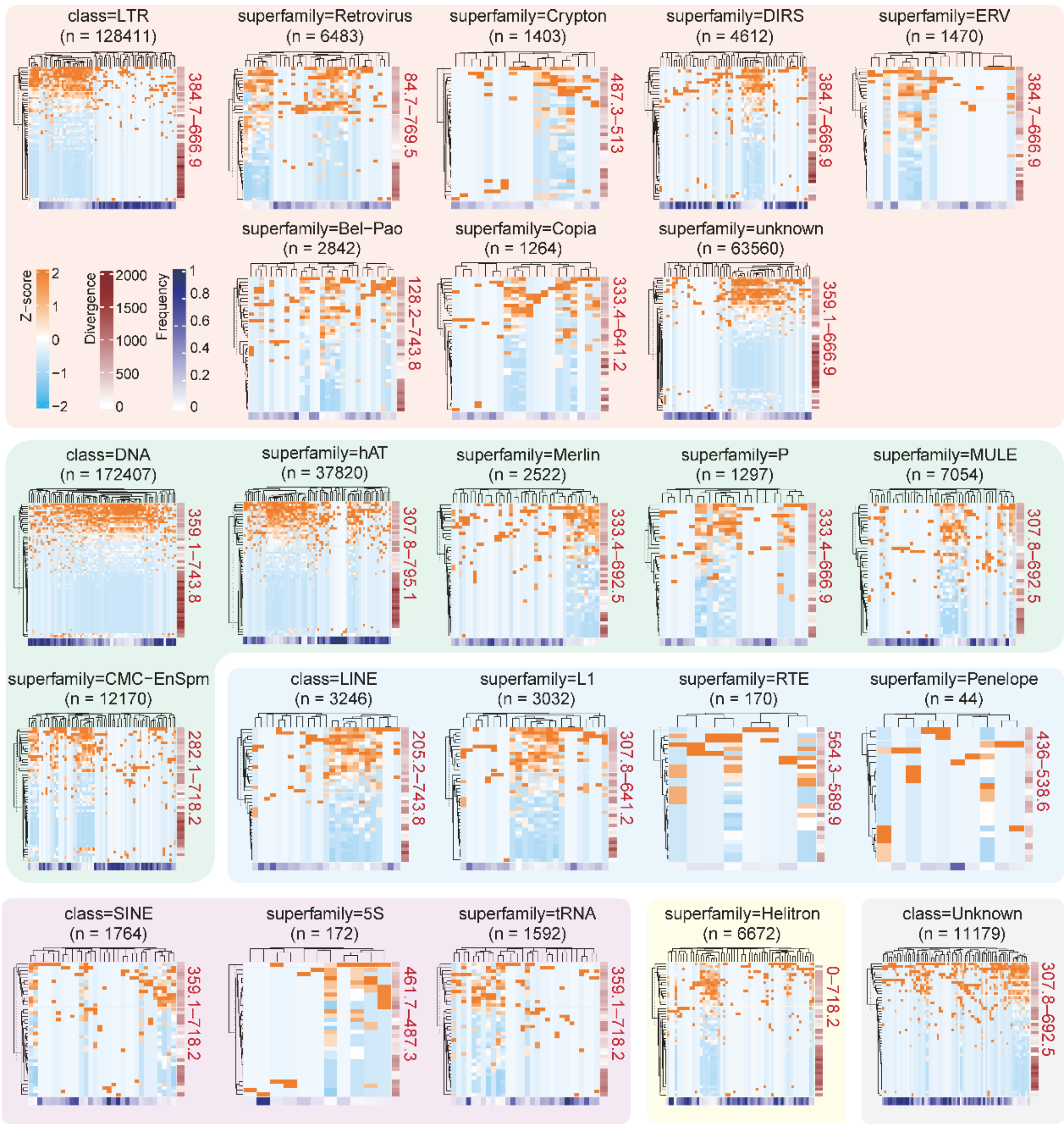

Figure S3. Heatmaps of TE insertion time and allele frequency in different TE superfamilies.

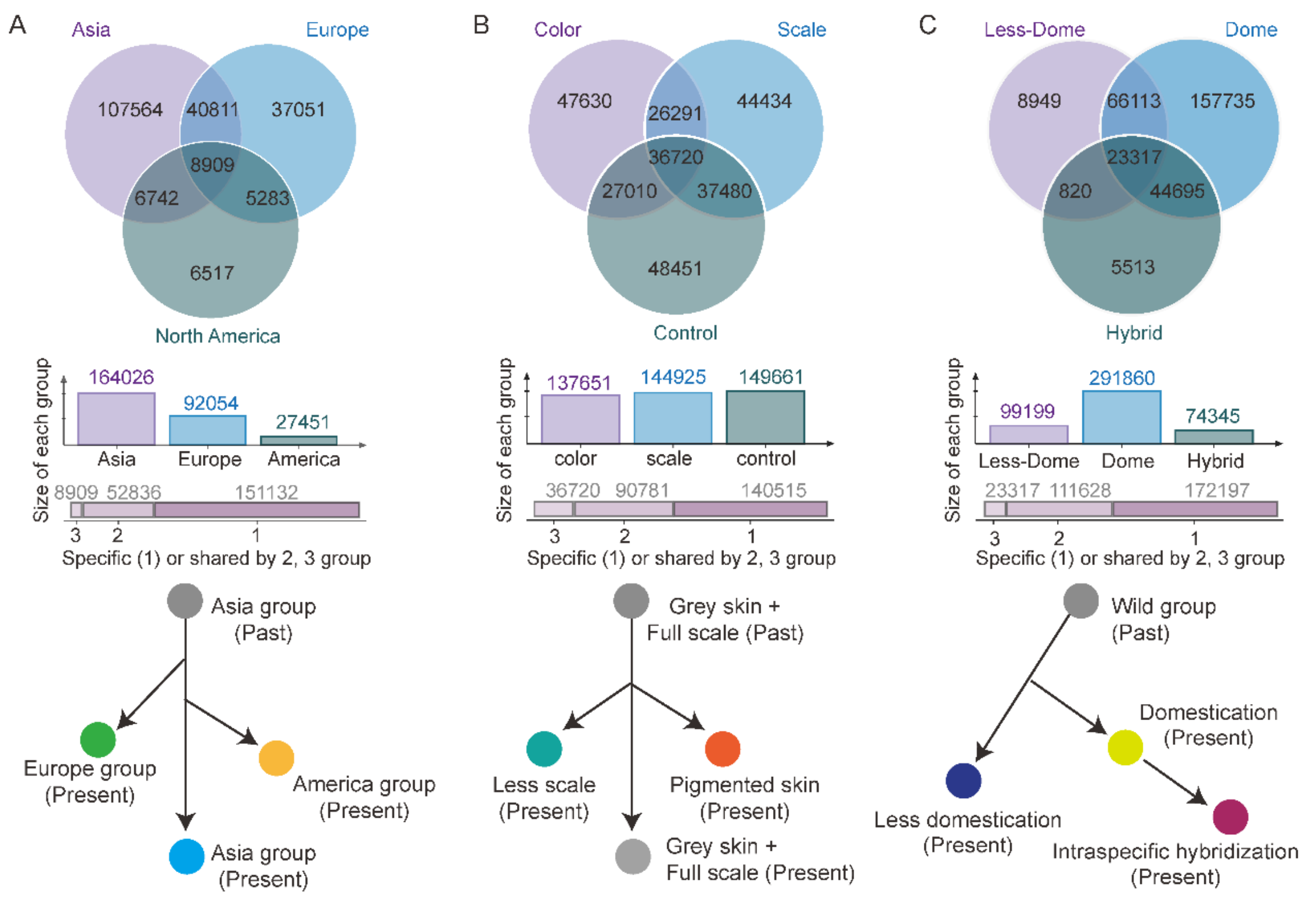

Figure S4. Comparison of TE distribution across geographical distribution, phenotypic traits, and domestication history. A) TE variant sharing across geographical groups. Venn diagram and bar plots show the number of specific and shared variants among groups from Asia, Europe, and North America. The evolutionary model depicts the radiation from the ancestral Asian group to present populations based on TE insertion distribution. B) TE variant distribution related to phenotypic traits. Overlap of variants among samples categorized by body color (pigmented skin), scale pattern (less scale), and control groups (grey skin + full scale). The model illustrates the transition from ancestral traits (grey skin + full scale) to divergent modern phenotypes. C) Distribution of TE variants among less-domesticated, domesticated, and hybrid groups. The model outlines the progression from wild ancestors to domesticated strains and subsequent intraspecific hybridization.

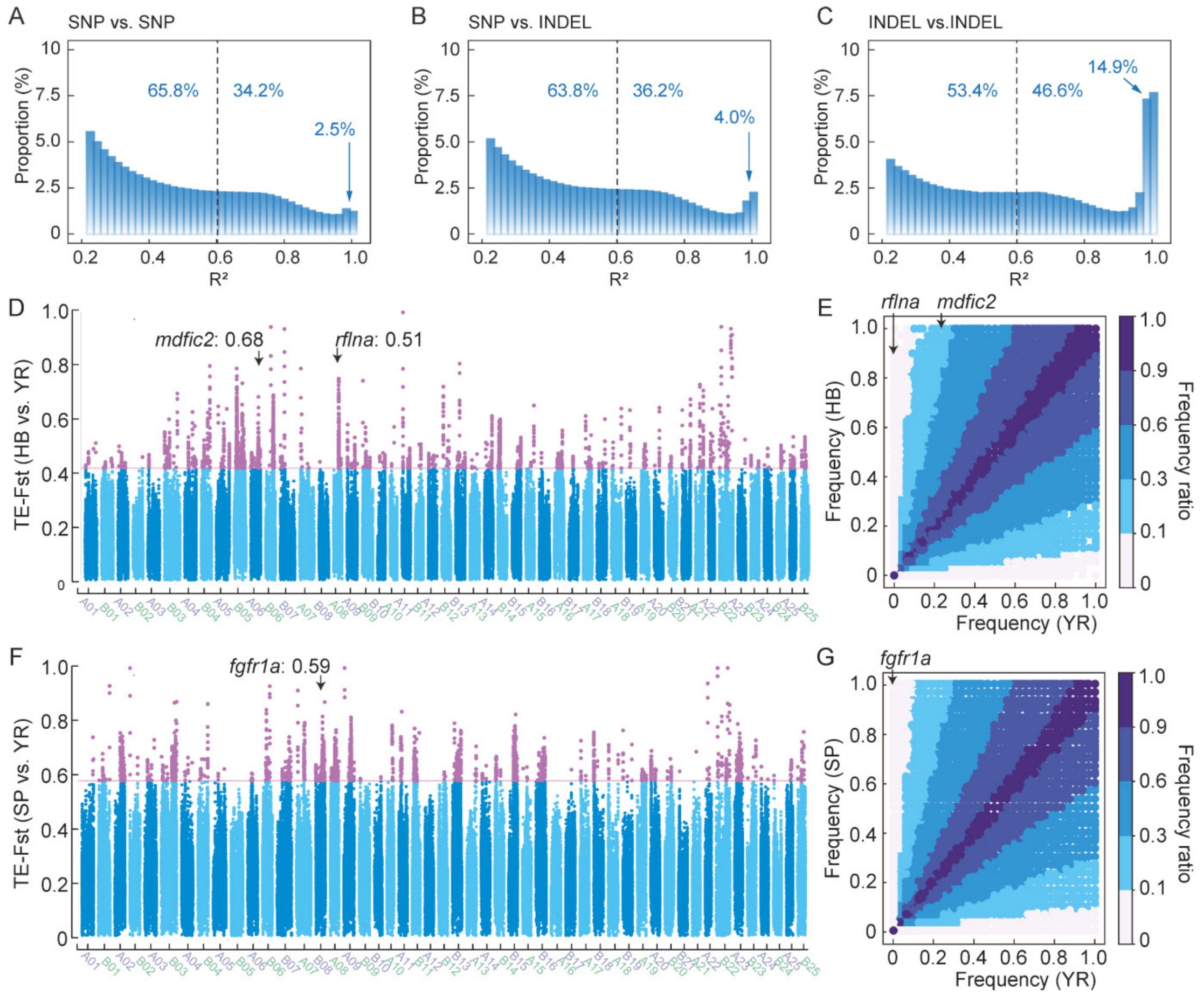

Figure S5. Linkage disequilibrium (LD) and population differentiation analysis. A-C) Distribution of linkage disequilibrium between variant types. (A) SNP vs. SNP, (B) SNP vs. indel, and (C) indel vs. indel. D) TE-Fst analysis between HB carp (red skin) and YR carp (grey skin). E) Allele TE frequency correlations between HB carp and YR carp. F) TE-Fst analysis between SP carp (less scale) and YR carp (full scale). The red line indicates the significance threshold. G) Allele TE frequency correlations between SP carp and YR carp. Color intensity represents the frequency ratio.

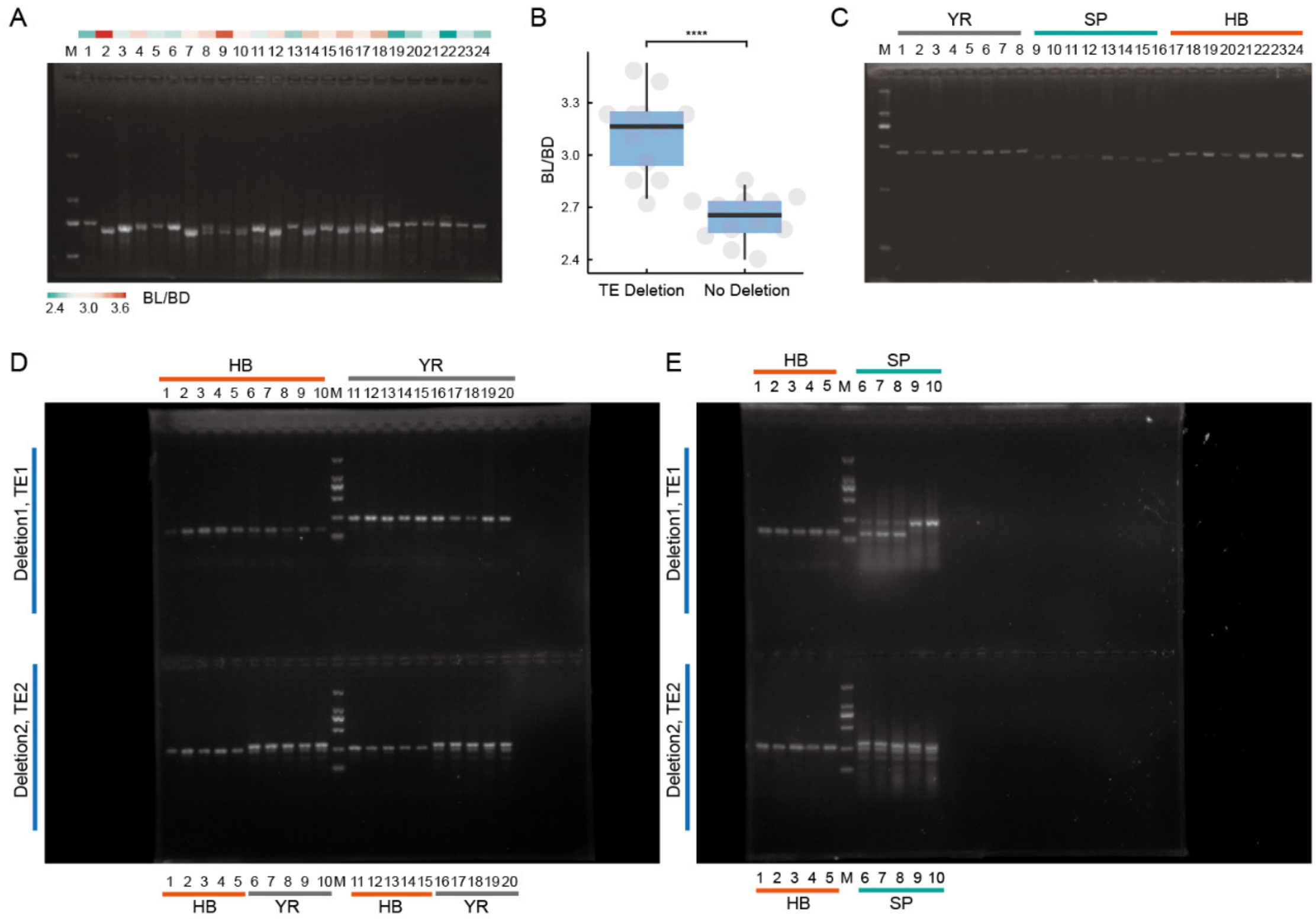

Figure S6. Original uncropped agarose gel images for PCR validations. A) PCR validation of the DNA/TcMar polymorphism at the *rflna* locus. Lane M represents the DNA marker, and from bottom to top: 500, 750, 1,000, and 2,000 bp. B) Comparison of BL/BD ratios between *rflna* deletion carriers and TE insertion carriers. Deletion carriers exhibited significantly higher BL/BD ratios than insertion carriers ****: $p < 0.0001$. C) PCR validation of the DNA/PIF-Harbinger deletion at the *fgfr1a* locus in three carp strains. The deletion was detected only in the scaleless SP strain but was absent from the scaled YR and HB strains. Lane M represents the DNA marker, and from bottom to top: 100, 250, 500, 750, 1,000, and 2,000 bp. D) Validation of TE variants at the *mdfic2* locus between HB strain and YR strain. E) Validation of TE variants at the *mdfic2* locus between HB strain and SP strain. Lane M represents the DNA marker, and from bottom to top: 100, 250, 500, 750, 1,000, and 2,000 bp.

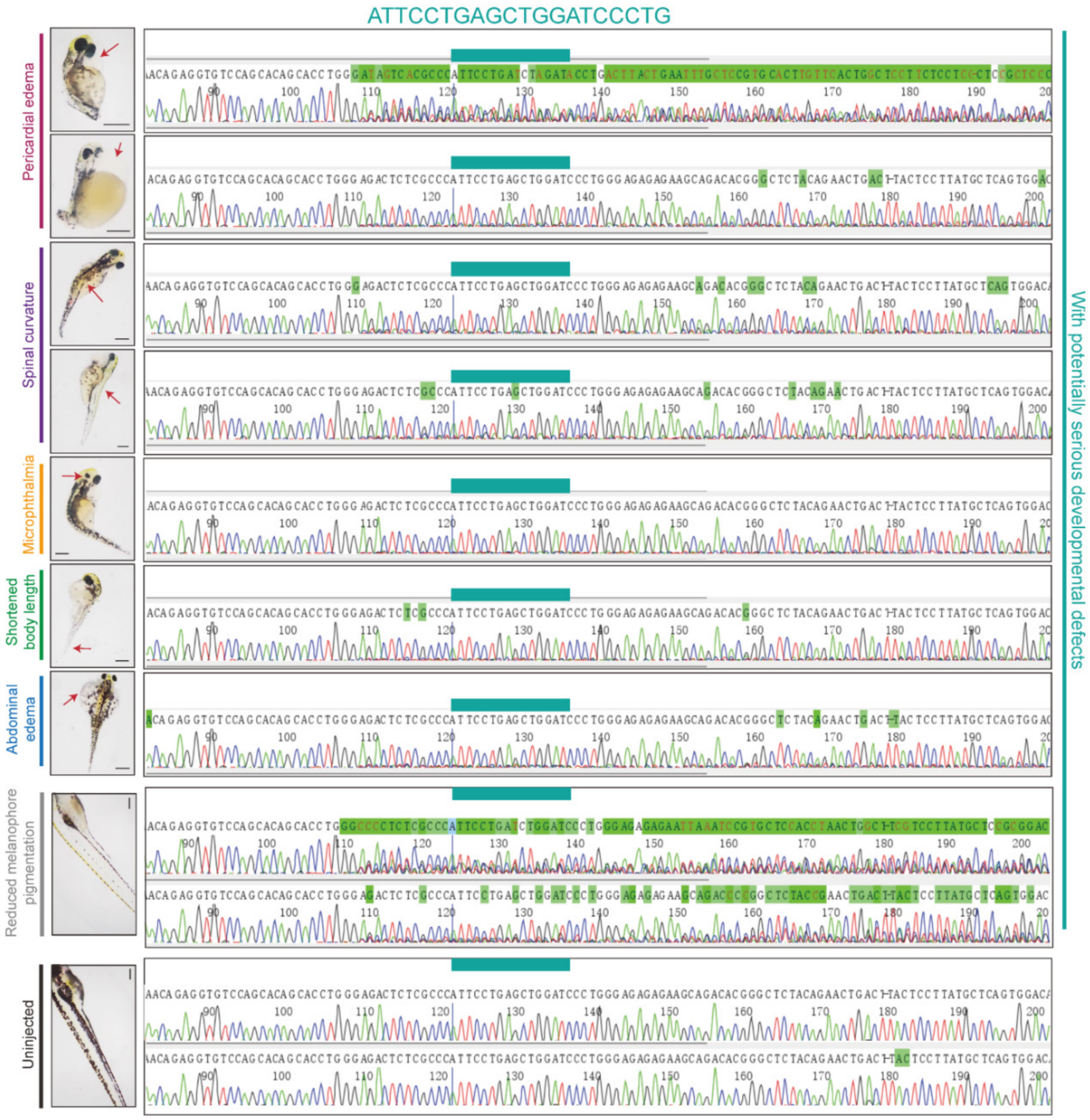

Figure S7. Sequencing of zebrafish developmental defect phenotypes. Corresponding Sanger sequencing chromatograms of the target region for each phenotypic sample are shown on the right. The green sequence at the top represents the wild-type target (ATTCCTGAGCTGGATCCCTG). Overlapping peaks or base substitutions in the chromatograms confirm various sequence rearrangements or mutations at the target locus. Uninjected wild-type embryos are shown at the bottom as negative controls, exhibiting normal morphology and clear, unambiguous sequencing peaks.

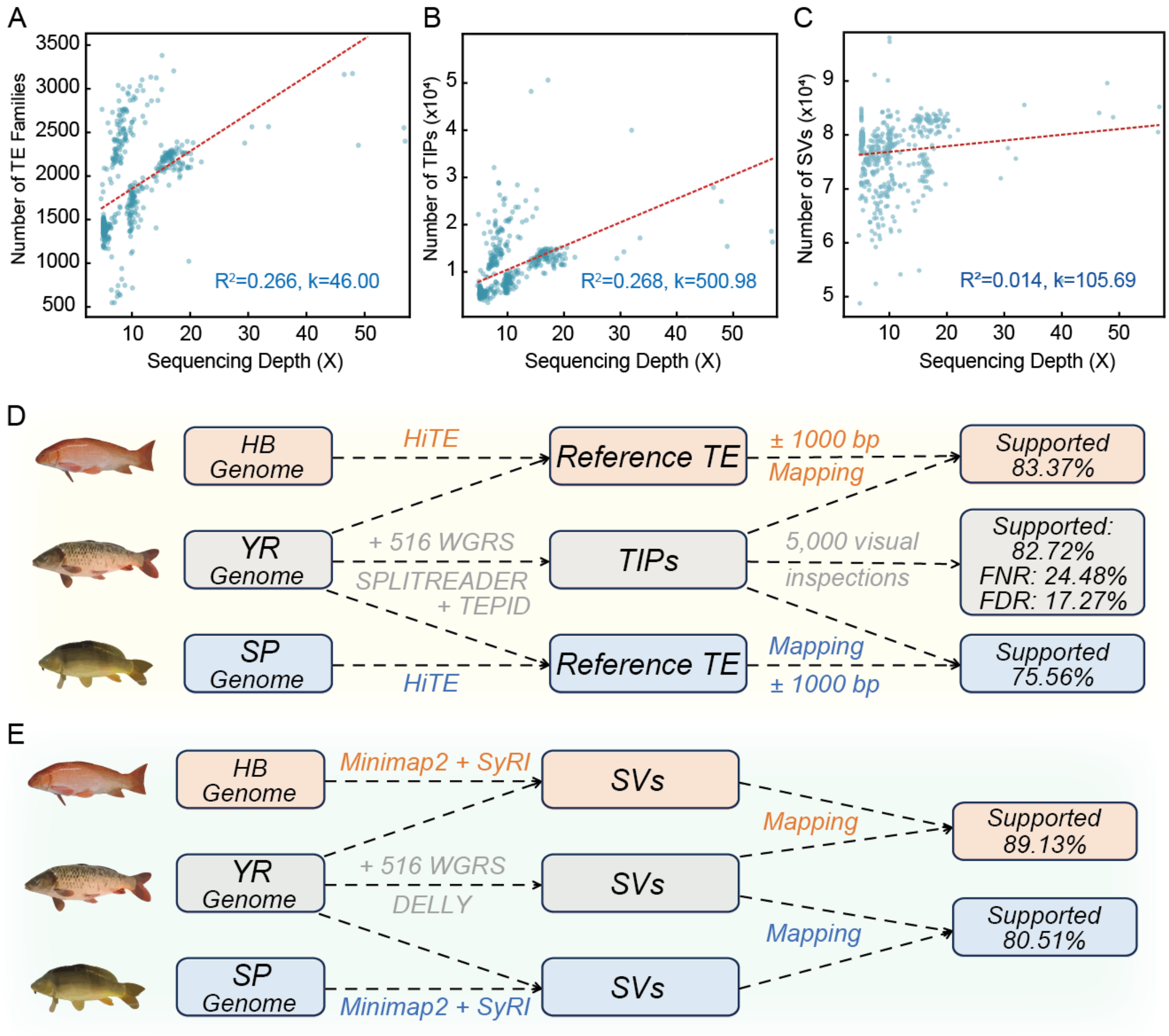

Figure S8. Assessment of TIP and SV calling reliability. (A–C) Numbers of TE families (A), TIPs (B), and SVs (C) across sequencing depths. Red dashed lines represent linear fits. (D) Independent validation of TIPs using chromosome-level genome assemblies and manual inspection. Reference TEs identified by HiTE in the HB and SP genomes were cross-mapped within ±1 kb of TIPs to assess support for population-level TE insertions. IGV manual inspection (n = 5,000, 200 randomly selected TIPs across 25 individuals) provided empirical estimates of false-negative and false-discovery rates. (E) Cross-genome validation of structural variants. SVs identified between the YR reference and HB/SP assemblies using Minimap2 and SyRI were cross-mapped against population-level SVs to assess support rates.
